# GSK3 maintains NSP12 stability to promote bat-origin swine acute diarrhea syndrome coronavirus infection

**DOI:** 10.1128/jvi.00683-26

**Published:** 2026-09-30

**Authors:** Maowen Sun, Cong Yuan, Yueyue Duan, Lei Shi, Yu Zhang, Luyao Li, Wenquan Mao, Haixue Zheng, Qi Wang

**Affiliations:** 1 State Key Laboratory of Animal Disease Control and Prevention, Lanzhou Veterinary Research Institute, Chinese Academy of Agricultural Sciences 111658, Lanzhou, China; Loyola University Chicago - Health Sciences Campus, Maywood, Illinois, USA

**Keywords:** swine acute diarrhea syndrome coronavirus, GSK3, NSP12, RdRp, RNA synthesis

## Abstract

Bat-origin swine acute diarrhea syndrome coronavirus (SADS-CoV) causes diarrhea and death in piglets and also poses a cross-species transmission risk due to its broad host cell tropism. In the absence of commercial vaccines or therapeutics, identifying the host factors exploited by SADS-CoV may provide potential targets for antiviral therapies. Inhibition of glycogen synthase kinase 3 (GSK3) restricts diverse coronavirus replication by targeting the N protein and disrupting its phosphorylation. Here, we identified GSK3 as a crucial host factor for SADS-CoV RNA synthesis. Both GSK3 deficiency and treatment with a classical GSK3 inhibitor, lithium chloride (LiCl), strongly impaired SADS-CoV dsRNA synthesis. LiCl treatment did not alter electrophoretic mobility or expression of SADS-CoV N protein in N-transfected Huh7 cells but significantly reduced NSP12 protein levels. The absence of GSK3 led to the proteasomal degradation of NSP12 to reduce viral RNA synthesis. However, GSK3 interacted with ubiquitin-specific peptidase 27X (USP27X) and promoted the USP27X-mediated deubiquitination of NSP12, thereby enhancing NSP12 stabilization and facilitating viral RNA synthesis. LiCl not only protected neonatal mice from lethal SADS-CoV challenge but also exhibited a notable broad-spectrum antiviral effect against PEDV and TGEV. Our findings demonstrate that GSK3-mediated NSP12 stabilization is essential for SADS-CoV RNA synthesis, underscoring the potential of GSK3 as a broad-spectrum antiviral target for emerging and re-emerging swine coronaviruses.

**IMPORTANCE:**

Zoonotic transmission of coronaviruses continues to pose a significant threat to human populations. Swine acute diarrhea syndrome coronavirus (SADS-CoV) has been identified as a potentially high-risk emerging coronavirus pathogen that could adversely affect the global economy and human health. However, the replication mechanisms of SADS-CoV remain largely unexplored. Our study demonstrates that GSK3 stabilizes NSP12 by interacting with USP27X, thereby enhancing the synthesis of SADS-CoV dsRNA. LiCl, a GSK3 inhibitor, exhibits broad-spectrum antiviral activity against swine enteric alphacoronaviruses. Our work is distinguished for the first time by the GSK3–USP27X regulatory axis that controls the stability of NSP12 and indicates that GSK3 is a promising therapeutic target for the development of broad-spectrum antiviral drugs against emerging or re-emerging swine coronaviruses.

## INTRODUCTION

Coronaviruses have caused pandemics worldwide among humans and animals, posing a serious threat to public health and food safety over the past two decades (1–3). Since the 21st century, three human coronaviruses, including severe acute respiratory syndrome coronavirus (SARS-CoV), Middle East respiratory syndrome coronavirus (MERS-CoV), and severe acute respiratory syndrome coronavirus 2 (SARS-CoV-2), have exhibited cross-species infection and caused severe respiratory illnesses as well as death around the world (2–7). SARS-CoV-2 is more transmissible than SARS-CoV or MERS-CoV and has caused over 14 million deaths based on excess mortality studies, which are significantly higher than the deaths previously reported by the World Health Organization (WHO) (2, 7–9). Coronaviruses predominantly cause respiratory diseases in humans and gastroenteritis in swine. Swine enteric coronaviruses (SeCoVs), such as transmissible gastroenteritis virus (TGEV) and porcine epidemic diarrhea virus (PEDV), have long been known to circulate in pigs, and they, together with novel identified porcine deltacoronavirus (PDCoV) and swine acute diarrhea syndrome coronavirus (SADS-CoV) in 2012 and 2017, respectively, cause watery diarrhea, vomiting, and death in piglets (10–15). Nevertheless, SeCoVs did not attract public attention until the emergence of a highly pathogenic PEDV variant strain in China in 2010, which caused a mortality rate of almost 100%, even in vaccinated suckling piglets (16).

From October 2016 to 2017, an unknown fatal diarrheal disease that was later identified as HKU2-related bat-origin SADS-CoV emerged in Guangdong, with a mortality rate as high as 90% in piglets under 5 days old (15, 17). The infection rates of SADS-CoV are lower than those of PEDV, TGEV, and PDCoV (18). However, the first outbreak of SADS-CoV was geographically close to that of SARS-CoV, and both originated from the same genus of horseshoe bats (17). The emergence of SADS-CoV indicates that bat-origin coronaviruses have expanded their host range to pigs (19). Besides swine cell lines, various cell lines of humans, bats, and monkeys are highly susceptible to SADS-CoV (20–22). However, SADS-CoV does not utilize known coronavirus receptors for its entry, suggesting the involvement of an uncharacterized receptor (20, 21). More importantly, SADS-CoV is lethal to neonatal mice, and diverse SADS-related CoVs (SADSr-CoV) from bats exhibit the potential for interspecies infection (23–25). Furthermore, coinfection and recombination of coronaviruses, well-documented drivers of viral evolution and the emergence of novel strains, have been observed in SADS-CoV (18, 26). All of these indicate that the host range of SADS-CoV may not be restricted to pigs, and it has the potential to expand to humans or other animals, highlighting the cross-species transmission and zoonotic risks of SADS-CoV.

SADS-CoV was identified nearly 10 years ago, but no vaccine or antiviral drug has been approved against SADS-CoV. The pathogenesis mechanism of SADS-CoV and its potential drug targets are rarely investigated. Only a few host factors involved in SADS-CoV infection have been identified, including zinc finger DHHC-type palmitoyltransferase 17 (ZDHHC17), transmembrane protein 53 (TMEM53), lymphocyte antigen 6 complex, locus E (LY6E), and poly(A)-binding protein 4 (PABPC4) (27–29). It has successfully identified critical host factors exploited by coronaviruses, influenza virus, and flavivirus infection in recent years, which offer potential targets with a broad-spectrum antiviral effect (30–33). Therefore, there is an urgent need to understand the pathogenesis mechanism of SADS-CoV and to attempt to develop an anti-SADS-CoV therapeutic using a host-targeted strategy with broad-spectrum antiviral activity.

Glycogen synthase kinase 3 (GSK3), a serine/threonine protein kinase, has two different isoforms, GSK3α and GSK3β, with overall 85% homology and 95% identity in catalytic domains (34). GSK3 favors influenza virus, hepatitis C virus, SARS-CoV, and SARS-CoV-2 infection (35–41). GSK3 phosphorylates the N protein of mouse hepatitis virus (MHV) JHM strain, and then the phosphorylated N protein can recruit the RNA helicase DDX1 for transition from discontinuous to continuous transcription, which is beneficial for long subgenomic RNA production (39). GSK3 inhibitors also possess promising therapeutic activity against influenza-like diseases *in vivo* and several coronaviruses, including SARS-CoV, MHV, PEDV, PDCoV, and SARS-CoV-2 *in vitro* (37–42). Our study identifies a previously uncharacterized role of GSK3 in SADS-CoV infection, which is distinct from its function in regulating N protein phosphorylation in coronaviruses (37–41). We demonstrate that GSK3 enhances SADS-CoV RNA synthesis and facilitates viral replication through promoting USP27X-mediated deubiquitination of NSP12, thereby stabilizing the NSP12 protein. Notably, the GSK3 inhibitor not only confers protection against lethal SADS-CoV challenge but also exhibits broad-spectrum antiviral activity against PEDV and TGEV. Collectively, these findings highlight a novel role of GSK3 in coronavirus replication and establish GSK3 as a promising therapeutic target for the development of broad-spectrum antiviral drugs against emerging or re-emerging swine coronaviruses.

## MATERIALS AND METHODS

### Cells and viruses

Huh7, HEK293T, Vero, and PK15 cells were cultured in Dulbecco’s modified Eagle medium (DMEM, Gibco) with 10% fetal bovine serum (FBS, Gibco) at 37°C. The SADS-CoV GDS04 strain (GenBank accession no. MF167434.1), the PEDV CV777 strain (GenBank accession no. AF353511.1), and the TGEV HLJ-17 strain (GenBank accession no. MT522161.1) were propagated and titrated on Huh7, Vero, or PK15 cells, respectively. Cells were inoculated with the indicated virus in serum-free medium. At 2 hours post-infection (hpi), the inoculum was discarded, and cells were cultured in fresh serum-free medium for the indicated time periods. All viruses were stored at −80°C in our laboratory.

### Plasmids and antibodies

The human *GSK3A* and *GSK3B* genes were synthesized from Sangon Biotech (Shanghai, China), cloned into p3xFlag-CMV-10 (Flag-GSK3α and Flag-GSK3β), and then subcloned into pCAGGS-HA (HA-GSK3α and HA-GSK3β) or pLVX-IRES-Puro (His-GSK3α and His-GSK3β). GSK3α and GSK3β mutant plasmids (S21A and S9A, respectively) were cloned into p3xFlag-CMV-10 (Flag-GSK3αS21A and Flag-GSK3βS9A) from Tsingke Biotech (Beijing, China) and subcloned into pLVX-IRES-Puro (His-GSK3αS21A and His-GSK3βS9A). The plasmids of Flag-USP22, -USP27X, -USP48, and -UCHL3 were purchased from MailGene Biotech (Beijing, China). The SADS-CoV *NSP12* gene and its mutants were amplified from SADS-CoV cDNA and cloned into p3xFlag-CMV-10 (Flag-NSP12, Flag-NSP12-RdRp, and Flag-NSP12-△RdRp). The SADS-CoV *NSP7* or *N* gene was amplified from SADS-CoV cDNA and cloned into the pEGFP-C2 vector (pEGFP-NSP7) or pCAGGS-HA vector (pHA-N), respectively. All primers used in the study are listed in Table 1. The rabbit antibody against the SADS-CoV N protein and the murine monoclonal antibody (mAb) against the PEDV N or TGEV N protein were made in-house. The HRP-conjugated GAPDH mAb (HRP-60004), antibodies against HA (51064-2-AP), Flag (20543-1-AP), and His (10001-0-AP) were purchased from Proteintech. Anti-dsRNA, clone rJ2 mAb (9Q04SL), or GFP-tag rabbit antibody (T0006) was obtained from Sigma-Aldrich or Affinity, respectively. Anti-GSK3 alpha/beta mAb (44610), the secondary antibody, including Goat anti-mouse IgG (H+L) Alexa Fluor 488 (A-11001), goat anti-rabbit IgG (H+L), Alexa Fluor 488 (A-11008), goat anti-mouse IgG (H+L) cross-adsorbed secondary antibody, Alexa Fluor 594 (A-11005), and goat anti-rabbit IgG (H+L) Alexa Fluor 594 (A-11012), or goat anti-rabbit IgG (H+L) antibody (HRP) (31460) were purchased from Thermo Fisher Scientific. Anti-phospho-glycogen synthase mAb (3891) was purchased from Cell Signaling Technology. Goat anti-mouse IgG (H+L) antibody (HRP) (ab205719) was purchased from Abcam.

**TABLE 1:** Primers used in the study

Primer	Sequence (5′−3′)
qGAPDH-F	GGAGCGAGATCCCTCCAAAAT
qGAPDH-R	GGCTGTTGTCATACTTCTCATGG
qSADS-CoV-N-F	CTAAAACTAGCCCCACAGGTC
qSADS-CoV-N-R	TGATTGCGAGAACGAGACTG
qGSK3A-F	AAGGTTCTCCAGGACAAG
qGSK3A-R	AAGTATCTCAGCCTCACAA
qGSK3B-F	TAATCTGGTGCTGGACTA
qGSK3B-R	CTGATACATATACAACTTGACAT
pEGFP-NSP7-F	TCAAGCTTCGAATTCATGTCCAAACTTACC
pEGFP-NSP7-R	AGATCCGGTGGATCCTTACTGCAGCACATT
pHA-N-F	GATTACGCTGAATTCATGGCCACTGTTAAT
pHA-N-R	ATCTGCTAGCTCGAGCTAATTAATAATCTC
pHis-GSK3α-F	ATTTCCGGTGAATTCATGAGCGGCGGCGGA
pHis-GSK3α-R	AGAGGGGCGGGATCCCTAGTGGTGGTGGTG GTGGTGGCCGCCGCTGCTATTGGTCAGG
pHis-GSK3β-F	ATTTCCGGTGAATTCATGAGCGGCAGGCCC
pHis-GSK3β-R	AGAGGGGCGGGATCCCTAGTGGTGGTGGTG GTGGTGGCCGCCTGTGGAGTTGCTGGC
pHA-USP27X-F	GATTACGCTGAATTCATGTGTAAGGACTAT
pHA-USP27X-R	ATCTGCTAGCTCGAGTCAGTAGGCTTGTGT
pHA-USP27X (C87A)-F	TCTTGGCAACACGGCCTTTATGAACTGCAT
pHA-USP27X (C87A)-R	GGCCGTGTTGCCAAGATT
pFlag-NSP12-F	GCGGCCGCGAATTCAATGCTGGAGCCTTGC
pFlag-NSP12-R	TGCCACCCGGGATCCTTACTGCAGCACGGT
pFlag-NSP12-△RdRp-R	TGCCACCCGGGATCCTTACAGCCGGGAGTTGTG
pFlag-NSP12-RdRp-F	GCCGCGAATTCAATGAGCATGACCGACCTG
qTGEV-N-F	CAATTCCCGTGGTCGGAAGA
qTGEV-N-R	TTTACGTTGGCCCTTCACCA
qGAPDH(Pig)-F	GAAGGTCGGAGTGAACGGATTT
qGAPDH(Pig)-R	TGGGTGGAATCATACTGGAACA
qPEDV-N-F	TGTAGTTGAGATTGTTGA
qPEDV-N-R	TTATTGCCTCTGTTGTTA
qGAPDH(Monkey)-F	CTCTGGTAAAGTGGATATTG
qGAPDH(Monkey)-R	GGTGGAATCATACTGGAA
qPEDV-M-F	GATACTTTGGCCTCTTGTGT
qPEDV-M-R	CACAACCGAATGCTATTGACG
PEDV-probe	TTCAGCATCCTTATGGCTTGCATC

### Western blotting

Cells were transfected with plasmids or infected with the virus at an indicated MOI. The cells were lysed in NP40 lysis buffer (Beyotime, P0013F) supplemented with 1% PMSF (Solarbio, P0100) for 15 min at 4°C, and then the cell lysates were centrifuged for 15 min at 4°C. Total proteins were boiled with loading buffer (Biosharp, BL502A) for 10 min and separated by SDS-PAGE. Separated proteins were transferred onto a nitrocellulose filter (NC) membrane (Pall, 66485), and the NC membrane was blocked with 5% Difco Skim Milk (BD Difco, 232100) for 2 h at room temperature. The membrane was incubated with the indicated antibody overnight at 4°C and then incubated with HRP-conjugated secondary antibodies. Protein signals were measured by incubating the NC membrane with the WesternBright ECL kit (Advansta, K-12045-D50).

### Co-immunoprecipitation assay

HEK293T cells were transfected with the indicated plasmids, and cell lysates were incubated at 4°C overnight using HA-beads (Thermo Fisher Scientific, 88837) or Flag-beads (Sigma, M8823). After washing with PBS supplemented with 1% NP40 lysis buffer five times, the beads were resuspended in the loading buffer and boiled for 10 min. The IP samples were subjected to western blotting and incubated with the indicated antibodies.

### RT-qPCR assay

Cells were infected with the virus at an indicated MOI or transfected with the indicated plasmids or small interfering RNA (siRNA). Total RNA was extracted from the cells using RNAiso Plus (Takara, 9108Q) and transcribed into cDNA by PrimeScript RT Master Mix (Takara, RR036A). The cDNA was used as a template for RT-qPCR analysis using ChamQ Universal SYBR qPCR Master Mix (Vazyme, Q71103) to detect the interference efficiency of siRNA or measure the viral *N* gene transcription level. The *GAPDH* gene was served as a normalization control. The relative gene level was quantified using 2^−(△△CT)^ method.

For absolute quantification of viral genomic copy numbers, 0.5 µg of RNA was served as a template for cDNA synthesis. Genomic copy numbers of SADS-CoV or TGEV were detected using SYBR Green-based absolute RT-qPCR with *N* gene-specific primer pairs combined with ChamQ Universal SYBR qPCR Master Mix. For detection of PEDV genomic RNA, a TaqMan probe-based absolute RT-qPCR assay was performed using PEDV *M* gene-specific primer pairs and probe, together with Probe qPCR Mix (TaKaRa, RR391A).

To distinguish and detect the positive-sense viral RNA (+vRNA) and negative-sense viral RNA (−vRNA) of SADS-CoV, +vRNA or −vRNA was obtained using the PrimeScript RT Reagent Kit with gDNA Eraser (Takara, RR047A) with SADS-CoV *N*-specific reverse or forward primer, respectively. The cDNA was subjected to SYBR Green-based absolute RT-qPCR with SADS-CoV *N*-specific primer pairs. Primers and probe used in the RT-qPCR assay were listed in Table 1.

### RNA interference assay

The human *GSK3A* or *GSK3B* gene was knocked down by siRNAs using the RNA sequences listed in Table 2. Huh7 cells were seeded in 24-well plates for 24 h at 37°C with 5% CO_2_. The cells were transfected with 50 nM siGSK3A, siGSK3B, a mixture of siGSK3A and siGSK3B, or siNC using the jetPRIME transfection reagent (Polyplus, 101000046) according to the manufacturer’s instructions. The transcription or expression level was determined by RT-qPCR or western blotting, respectively.

**TABLE 2:** Small interfering RNA targeting GSK3 used in the study

Name	Sense sequence (5′−3′)	Antisense sequence (5′−3′)
hGSK3A-1196	GUUCAAGUUCCCUCAGAUUAATT	UUAAUCUGAGGGAACUUGAACTT
hGSK3A-496	CGGACAUCAAAGUGAUUGGCATT	UGCCAAUCACUUUGAUGUCCGTT
hGSK3A-635	GCUGGACCACUGCAAUAUUGUTT	ACAAUAUUGCAGUGGUCCAGCTT
hGSK3B-2073	GAUGAAUUACGGGACCCAAAUTT	AUUUGGGUCCCGUAAUUCAUCTT
hGSK3B-1277	GCAGGACAAGAGAUUUAAGAATT	UUCUUAAAUCUCUUGUCCUGCTT
hGSK3B-1868	CCCAAACUACACAGAAUUUAATT	UUAAAUUCUGUGUAGUUUGGGTT

### CRISPR-Cas9 knockout assay

To construct the *GSK3A*- or *GSK3B*-KO Huh7 cell line, sgRNAs targeting GSK3A (5′-GCCCGAGACAGTGTACCGGG-3′) and GSK3B (5′-AGATGAGGTCTATCTTAATC-3′) were synthesized and cloned into the lentiCRISPR V2 vector after *Bsm*bI-digestion, named lentiCRISPR V2-GSK3A or -GSK3B, respectively. Lentiviruses were produced by cotransfecting lentiviral helper plasmids (pMD2.G and pSPAX2) together with lentiCRISPR V2-GSK3A or -GSK3B into HEK293T cells using the jetPRIME transfection reagent. After 48 hours post-transfection (hpt), the cells were stored at −80°C and then centrifuged at 2,000 rpm for 5 min. The supernatant was filtered with a 0.45-µm filter (Merck, SLHV033RB), and the viruses were stored at −80°C. Huh7 cells were infected with the indicated lentiviruses carrying GSK3A or GSK3B sgRNAs for 48 h, respectively. The cells were selected with puromycin (1 µg/mL) for 2 weeks. Surviving cells were cultured and validated by western blotting.

### Cell viability assay

Cell viability of knockout or inhibitor-treated cells was measured by Cell Counting Kit-8 (CCK-8) (Beyotime, C0039) following the manufacturer’s instructions. The cells were seeded in 96-well plates and cultured for 24 h or treated with the inhibitor for an additional 24 h. The cells were treated with CCK-8 reagents for 4 h, and the absorbance was recorded.

### Viral attachment and internalization assay

For the attachment assay, Huh7 cells were first transfected with siRNA, or KO cells were cultured for 24 h, and then incubated with SADS-CoV for 1 h at an MOI of 1 at 4°C. The supernatant was removed, and the cells were washed with phosphate-buffered saline (PBS) three times. Total RNA was extracted, and viral genomic copy numbers were detected by RT-qPCR. For the internalization assay, the cells were cultured as described above, followed by incubation for another 1 h at 37°C. After three washes to remove unbound virions, the cells were lysed in RNAiso Plus reagent for RNA extraction and detection of viral RNA levels using RT-qPCR.

### Inhibitor assay

Huh7 cells were seeded into a 24-well plate and cultured for 24 h. The cells were infected with SADS-CoV at an MOI of 1 and incubated for 2 h at 37°C with 5% CO_2_. The cells were washed twice with PBS and cultured in the presence or absence of lithium chloride (LiCl) (APExBIO, B6083). Total RNA or cell lysates were extracted from cells at 0, 2, 4, 6, 8, and 12 hpt for RT-qPCR or western blotting, respectively.

### Laser confocal microscopy

The knockdown or knockout of GSK3 cells was cultured for 24 h and infected with SADS-CoV at an MOI of 1 at 37°C. After 2 hpi, the cells were washed with PBS and collected at 8, 12, or 24 hpi. The cells were fixed with 4% paraformaldehyde (Biosharp, BL539A) and then permeabilized with 0.3% Triton X-100. After washing three times with PBS, the cells were incubated with mouse anti-dsRNA mAb followed by overnight incubation with Alexa Fluor 488-conjugated goat anti-mouse IgG (H+L) antibody at 4°C. Cells’ nuclei were stained with 4′,6-diamidino-2-phenylindole (DAPI) (Solarbio, C0060) for 10 min at room temperature. After washing three times, cells were observed by laser confocal microscopy. For the effect of LiCl on dsRNA, Huh7 cells were infected with SADS-CoV (MOI = 1) for 2 h. After washing three times with PBS, the cells were treated with LiCl for 8, 12, and 24 h. The amounts of dsRNA in the LiCl-treated Huh7 cells were determined by anti-dsRNA as described above.

For the subcellular localization of GSK3 and SADS-CoV dsRNA, Huh7 cells were transfected with pFlag-GSK3β or an empty vector for 24 h and then infected with SADS-CoV at an MOI of 1. After 24 hpi, the cells were fixed and incubated with rabbit antibody against Flag or GSK3α and mouse anti-dsRNA mAb. Following incubation with Alexa Fluor 594-conjugated goat anti-rabbit IgG (H+L) antibody or Alexa Fluor 488-conjugated goat anti-mouse IgG (H+L) antibody, the nuclei were stained with DAPI as described above. Subcellular localization of GSK3α or GSK3β and dsRNA was observed using laser confocal microscopy.

For the subcellular localization of GSK3 and NSP12, HEK293T cells were transfected with pHA-GSK3α, pHA-GSK3β, or pFlag-NSP12 individually or together for 24 h. HEK293T cells were fixed and incubated with a rabbit antibody against HA or a mouse antibody against Flag. Subsequently, the cells were incubated with the indicated secondary antibody, and the cellular localization was described above.

### RNA-dependent RNA polymerase reporter assay

To evaluate and reflect SADS-CoV RdRp activity, we cloned sequences harboring the sense *Renilla* luciferase (*Rluc*) gene and the antisense *firefly* luciferase (*Fluc*) gene, flanked by the antisense 5′ untranslated region (5′-UTR) and 3′-UTR of SADS-CoV, with the hepatitis delta virus (HDV) ribozyme elements positioned on both sides of the 5′-UTR and 3′-UTR, into the pCDNA3.1 vector. We constructed a cell-dependent RdRp-reporter plasmid (pCDNA3.1-RdRp reporter plasmid) by modifying a similar reporter system for HCV as described previously (43). HEK293T cells were transfected with siRNA targeting GSK3 or a negative control for 24 h and then transfected with the RdRp reporter plasmid together with pFlag-NSP12 or an empty vector for another 24 h. The cell lysates were collected, and the expression of Fluc and Rluc was determined using the Nano-Glo Dual-Luciferase Reporter Assay System (Promega, N1610). The SADS-CoV RdRp activity is represented by the ratio of Fluc to Rluc.

### Animal experiments

Two-day-old BALB/c neonatal mice were obtained from the Lanzhou Veterinary Research Institute, Chinese Academy of Agricultural Sciences. The neonatal mice were divided into four groups, with two groups being orally inoculated with 10^3^ TCID_50_ SADS-CoV GDS04 strain per mouse, while another group received an equal volume of DMEM. After 12 h, neonatal mice in the infected groups were administered LiCl orally at doses of 4 mg/kg body weight or an equal volume of PBS for 6 consecutive days. Uninfected mice were orally administered the same doses of LiCl or an equal volume of PBS as described above. The body weight and survival rate of the neonatal mice were recorded daily for 7 days after SADS-CoV challenge. Surviving mice were euthanized, and tissues, including the intestine, liver, lung, and brain, were collected from all mice for viral load detection. The intestine was used for histopathological analysis.

### Statistical analysis

All data were taken from triplicates and analyzed using a *t*-test in GraphPad Prism 8.0.2. A *P* value <0.05, <0.01, <0.001, or >0.05 was represented as a significant difference (*), highly significant difference (**), extremely significant difference (***), or no significant difference (ns), respectively.

## RESULTS

### GSK3 is an essential gene for SADS-CoV replication

GSK3 has two different isoforms, GSK3α and GSK3β, with an overall 85% sequence identity (34). To verify the role of GSK3 in SADS-CoV infection *in vitro*, we screened a series of small-interfering RNA (siRNA) targeting the *GSK3A* or *GSK3B* gene (siGSK3A or siGSK3B) in Huh7 cells. We found that a > 70% reduction in the transcription level of the *GSK3A* or *GSK3B* gene was observed in the siGSK3A- or siGSK3B-transfected cells compared to the siRNA negative control (siNC) (Fig. S1A and B). Knockdown of GSK3 in Huh7 cells markedly reduced SADS-CoV genomic copy numbers by approximately 1.7-log and N protein levels (Fig. S1C through E). The siGSK3A or siGSK3B had great specificity (Fig. S1F and G), and knockdown of either *GSK3A* or *GSK3B* significantly inhibited SADS-CoV replication (Fig. S1H through J). Subsequently, we overexpressed GSK3α or GSK3β in Huh7 cells, followed by infection with SADS-CoV. At 24 hpi, a significant upregulation in the transcription level of the SADS-CoV *N* gene was observed, along with an increase in the SADS-CoV N protein level (Fig. S1K and L).

**Fig 1:**
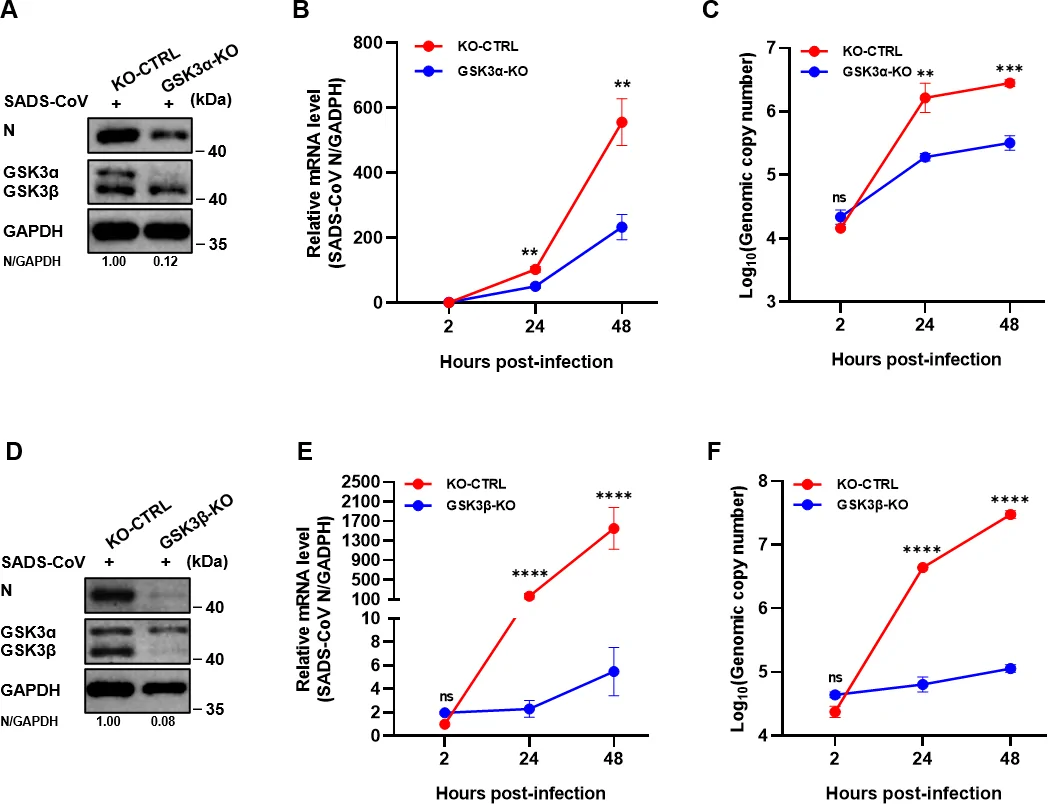
GSK3 is an essential gene for SADS-CoV replication. (**A**) The SADS-CoV N protein level in GSK3α knockout (GSK3α-KO) and control (KO-CTRL) cells was determined by western blotting at 24 hours post-infection (hpi) with SADS-CoV at an MOI of 1. (**B and C**) Viral *N* gene transcription levels (**B**) and genomic copy numbers (**C**) in GSK3α-KO cells were determined by RT-qPCR at 2, 24, and 48 hpi. (**D**) Western blotting assay was used to detect the viral N protein level in SADS-CoV-infected GSK3β-KO cells at 24 hpi. (**E and F**) Following incubation of SADS-CoV (MOI = 1) for 2, 24, and 48 h, viral *N* gene levels (**E**) and genomic copy numbers (**F**) in the GSK3β-KO cells were determined by RT-qPCR. Western blots were repeated at least three times, and one of them was selected as representative. Data are presented as mean ± SD, with statistical significance indicated as ns, *P* > 0.05, **P < 0.01, ***P < 0.001, and ****P < 0.0001, determined by a two-tailed *t*-test.

To further validate these discoveries, we established Huh7 CRISPR-Cas9 knockout (KO) cell lines of *GSK3A* and *GSK3B* (GSK3α-KO and GSK3β-KO, respectively). The result of the immunoblot assay showed that the SADS-CoV N protein in the GSK3α-KO or GSK3β-KO was significantly decreased compared to negative control cells (KO-CTRL) (Fig. 1A and D). To determine whether the effect of GSK3 on SADS-CoV replication was specific rather than influencing cell proliferation. The ability of cell proliferation was determined by the CCK-8 assay. Deletion of *GSK3A* or *GSK3B* did not affect cell viability (Fig. S2A). Subsequently, KO cells were infected with SADS-CoV at an MOI of 1, and RT-qPCR quantified viral genomic copy numbers and the *N* gene transcription level at 2, 24, and 48 hpi, respectively. No significant difference was observed at 2 hpi. However, viral *N* gene transcription levels (Fig. 1B and E) and genomic copy numbers (Fig. 1C and F) in the GSK3α-KO or GSK3β-KO cells were significantly lower than those in KO-CTRL cells at both 24 and 48 hpi. These results strengthened the finding that SADS-CoV replication is dependent on GSK3.

### GSK3 is required for SADS-CoV RNA synthesis

**Fig 2:**
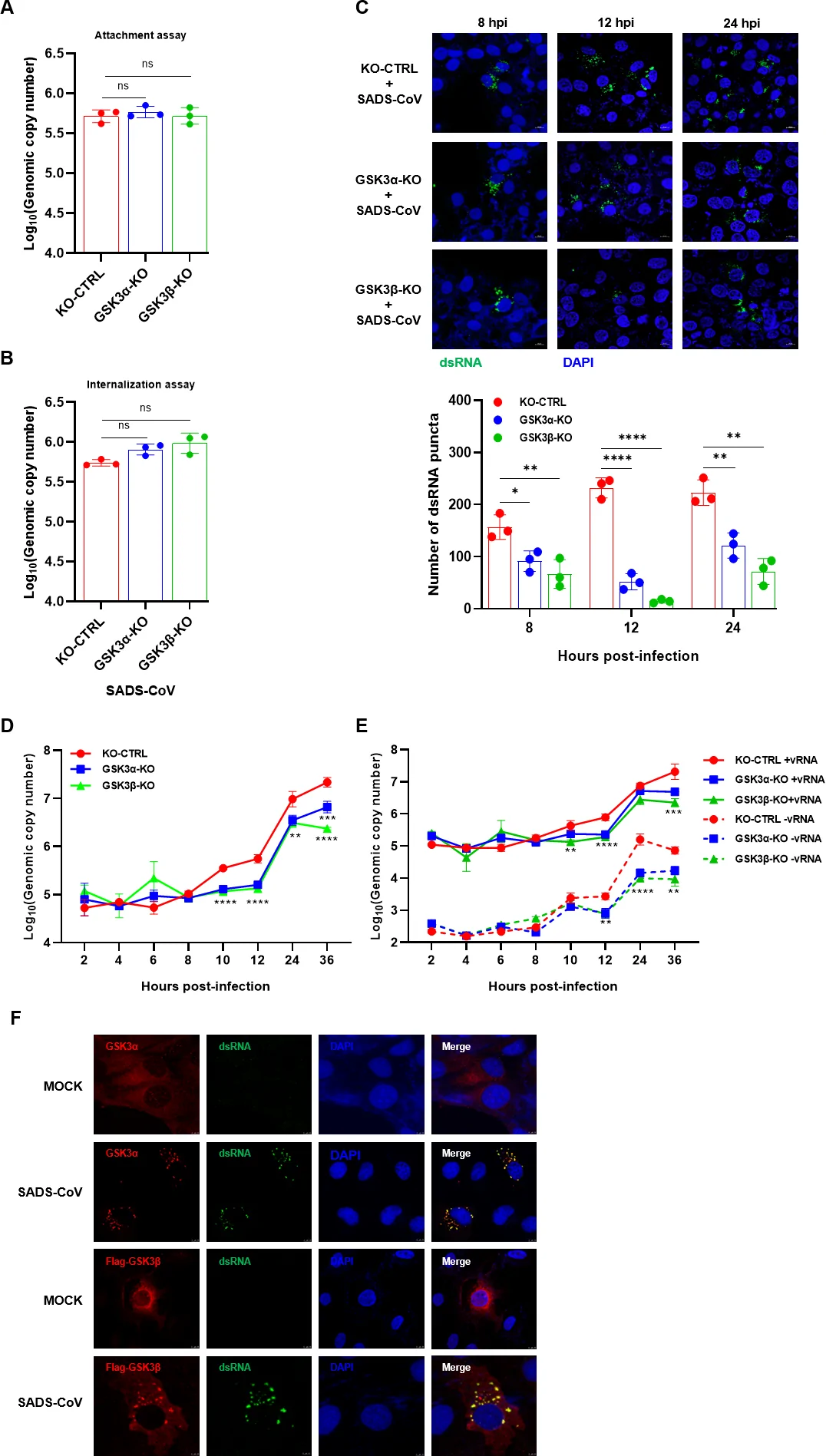
SADS-CoV RNA synthesis necessitates the involvement of GSK3. (**A and B**) The knockout of GSK3α or GSK3β does not affect viral attachment and internalization. KO-CTRL, GSK3α-KO, and GSK3β-KO cells were infected with SADS-CoV at an MOI of 1 for 1 h at 4°C (attachment assay) or subsequently incubated for an additional hour at 37°C (internalization assay). After three washes with PBS, total RNA was extracted to assess viral attachment (**A**) or internalization (**B**) using RT-qPCR. (**C**) Viral dsRNA (green) was stained using a mouse anti-dsRNA antibody at 8, 12, or 24 hpi. Scale bar, 10 µm. The quantification of viral dsRNA, performed using ImageJ software, is displayed in the lower panel. (**D and E**) Quantification of intracellular viral genomic RNA (**D**), as well as positive- and negative-stranded RNA (+vRNA or −vRNA) (**E**) in SADS-CoV-infected KO cells at 2, 4, 6, 8, 10, 12, 24, and 36 hpi, was conducted using RT-qPCR. (**F**) GSK3 is recruited to and colocalizes with viral dsRNA. Huh7 cells or pFlag-GSK3β-transfected Huh7 cells were infected with SADS-CoV, and subsequently, GSK3α (red), GSK3β (red), viral dsRNA (green), and cell nuclei (blue) were determined by staining with rabbit anti-GSK3α or -Flag antibodies in conjunction with mouse anti-dsRNA. Cell nuclei were stained with 4′,6-diamidino-2-phenylindole (DAPI) (blue) in confocal images. Scale bar, 10 µm. Data are expressed as the mean ± standard deviation (SD). Statistical significance is indicated as ns (*P* > 0.05), * (*P* < 0.05), ** (*P* < 0.01), *** (*P* < 0.001), and **** (*P* < 0.0001) based on a two-tailed *t*-test.

GSK3 inhibition impedes diverse coronavirus infection, but its role in the SADS-CoV lifecycle remains uncharacterized. Huh7 cells were transfected with siNC or siGSK3 for 24 h and then incubated with SADS-CoV for 1 h at 4°C or for an additional 1 h at 37°C at an MOI of 1, representing viral attachment or internalization, respectively. Virions on the cell surface or inside cells were quantified by RT-qPCR. No significant differences were observed in viral attachment and internalization, indicating that GSK3 did not affect SADS-CoV attachment and internalization (Fig. S3A and B). Double-stranded RNA (dsRNA) served as an intermediate in viral genome replication and subgenomic RNA (sgmRNA) and is generally recognized as a marker of viral RNA synthesis or replication organelles (ROs) in infected cells. Therefore, we detected dsRNA levels in the siGSK3-transfected Huh7 cells upon SADS-CoV infection. Knockdown of GSK3 resulted in a reduction in dsRNA accumulation at 8, 12, and 24 hpi (Fig. S3C). These results were further supported in the SADS-CoV-infected GSK3-KO cells (Fig. 2A through C). The knockout of GSK3 did not significantly affect viral attachment or internalization but led to a marked reduction in viral dsRNA during SADS-CoV replication (Fig. 2A through C). We also detected the total viral genomic RNA (gRNA) levels, as well as negative- or positive-stranded viral RNA (−vRNA or +vRNA) in SADS-CoV-infected KO cells at 2, 4, 6, 8, 10, 12, 24, and 36 hpi. In KO-CTRL cells, viral gRNA levels remained stable from 2 to 8 hpi and significantly increased from 8 to 36 hpi, while viral gRNA in the GSK3-KO cells significantly decreased at 10 hpi (Fig. 2D). The −vRNA and +vRNA were detected using specific forward- and reverse-strand primers, respectively. The results showed that the levels of −vRNA and +vRNA remained stable before 8 hpi and increased thereafter. However, their levels were lower in both GSK3α-KO and GSK3β-KO cells than in KO-CTRL cells, with significant reductions observed from 12 hpi and from 10 to 36 hpi, respectively (Fig. 2E). Interestingly, GSK3α and GSK3β were mainly localized in the cytoplasm in uninfected Huh7 cells, but GSK3α and GSK3β aggregated, were recruited to, and colocalized with dsRNA in SADS-CoV-infected cells (Fig. 2F). Therefore, the above results indicate that GSK3 is recruited to dsRNA and required for SADS-CoV RNA synthesis.

### The GSK3 inhibitor suppresses SADS-CoV RNA synthesis

**Fig 3:**
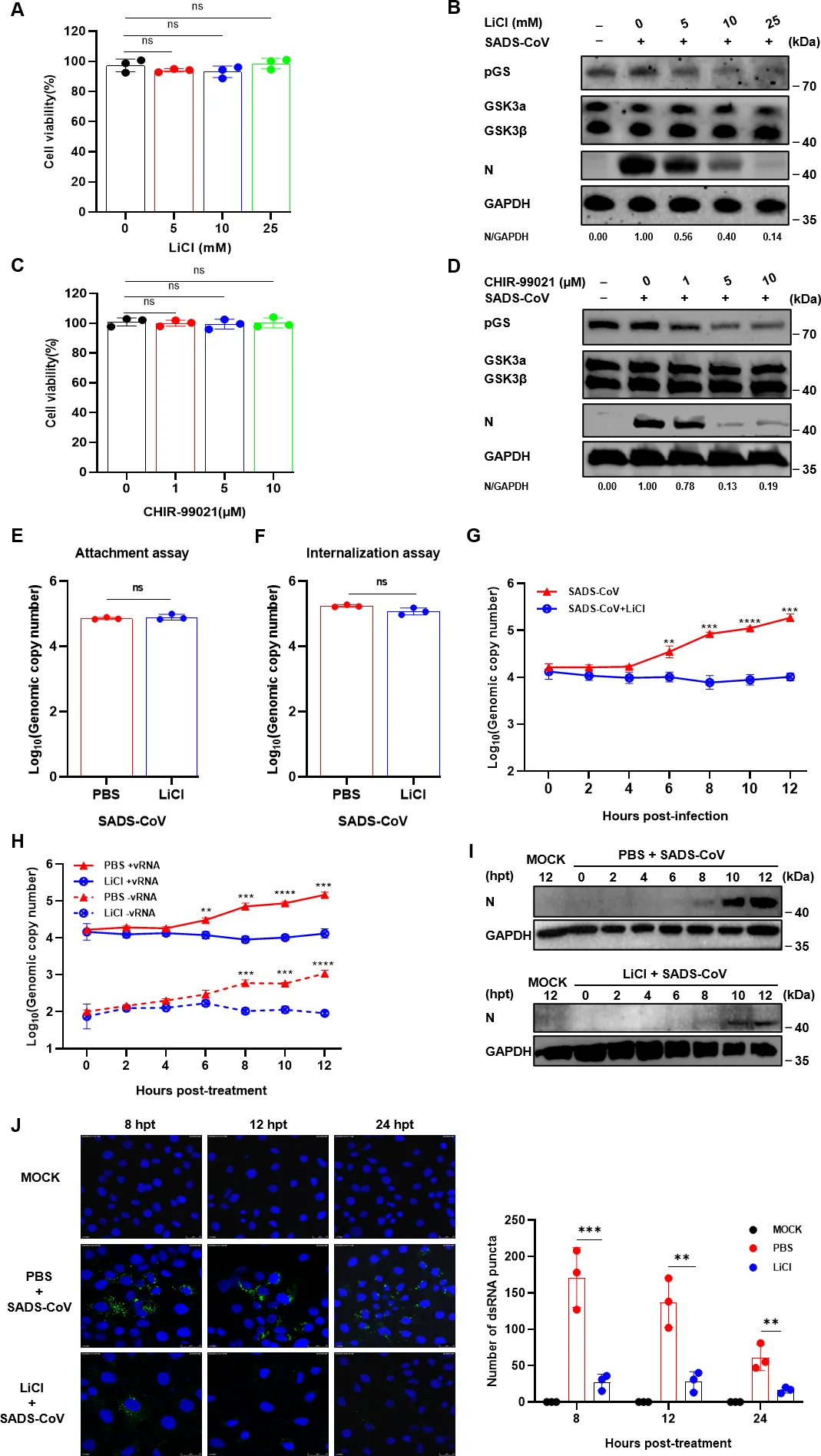
The GSK3 inhibitor effectively suppresses SADS-CoV RNA synthesis. (**A**) Huh7 cells were treated with LiCl at concentrations of 0, 5, 10, or 25 mM for 24 h, and cell viability was assessed using CCK-8 kits. (**B**) Huh7 cells were infected with SADS-CoV at an MOI of 1 for 2 h, followed by treatment with LiCl at concentrations of 0, 5, 10, or 25 mM. Viral N protein levels were detected by western blotting. (**C**) Cell viability of Huh7 cells in the presence of CHIR-99021 (0, 1, 5, 10 µM) was assessed by the CCK-8 kit. (**D**) Huh7 cells were infected with SADS-CoV (MOI = 1) for 2 h, followed by treatment with CHIR-99021 (0, 1, 5, and 10 µM). The expression levels of pGS, N, and GSK3 (both GSK3α and GSK3β) were evaluated through western blotting with rabbit anti-pGS, anti-SADS-CoV N, and mouse anti-GSK3 antibodies, respectively, with GAPDH serving as a loading control. (**E and F**) LiCl does not influence SADS-CoV attachment or internalization. For the attachment assay, Huh7 cells were incubated with SADS-CoV at an MOI of 1 in the presence or absence of 25 mM LiCl for 1 h at 4°C. For the internalization assay, Huh7 cells were exposed to SADS-CoV at an MOI of 1 for 1 h at 4°C, followed by culture for an additional hour at 37°C in the presence of LiCl (25 mM). The viral copy numbers from the attachment (**E**) and internalization (**F**) assays were determined using RT-qPCR. (**G–J**) LiCl disrupts SADS-CoV RNA synthesis. Huh7 cells were infected with SADS-CoV as previously described and then treated with 25 mM LiCl for 0, 2, 4, 6, 8, 10, and 12 h. Total RNA was extracted, and viral genomic copy numbers (**G**), positive- and negative-stranded RNA (+vRNA or −vRNA) (**H**), and N protein levels (**I**) in the cell lysate were quantified using RT-qPCR and western blotting with a rabbit anti-SADS-CoV N antibody. Following the LiCl treatment, viral RNA was stained with mouse anti-dsRNA mAb (green) and DAPI (blue) and visualized using laser confocal microscopy at 8, 12, or 24 h. Scale bar, 25 µm (**J**). Quantification of dsRNA was performed using ImageJ software (right panel). Western blots were repeated at least three times, and one of them was selected as representative. Statistical significance is indicated as ns (*P* > 0.05), * (*P* < 0.05), ** (*P* < 0.01), *** (*P* < 0.001), and **** (*P* < 0.0001) based on a two-tailed *t*-test.

Lithium chloride (LiCl) is a well-established and specific inhibitor of GSK3 that does not affect other known kinases (44). LiCl inhibits SADS-CoV replication without affecting cell viability in a dose-dependent manner (Fig. 3A and B). We also tested another GSK3 inhibitor, CHIR-99021, on SADS-CoV replication. Consistent with the results of LiCl, CHIR-99021 inhibited the SADS-CoV N protein level but did not affect cell proliferation (Fig. 3C and D). All of the results strongly indicate that the GSK3 inhibitor exhibits antiviral activity against SADS-CoV *in vitro*. We next tested the effect of LiCl on the SADS-CoV lifecycle by attachment and internalization assays as described, with a modification in Fig. 2. Huh7 cells were incubated together with SADS-CoV at an MOI of 1 and LiCl for 1 h at 4°C (attachment assay), or the cells were infected with SADS-CoV at 4°C, followed by incubation at 37°C for another 1 h with LiCl (internalization assay). Total RNA was extracted from the infected cells, and viral RNA levels were determined by RT-qPCR. No significant differences were observed in either attachment or internalization (Fig. 3E and F), consistent with Fig. 2, indicating that LiCl did not affect viral adsorption and entry. However, treatment with LiCl not only resulted in a reduction in viral gRNA from 6 hpt but also significantly inhibited the −vRNA and +vRNA levels from 8 hpt and 6 hpt, respectively (Fig. 3G and H). The SADS-CoV N protein was readily detectable from 8 hpt onward in the untreated cells, but its expression was hardly detectable in the presence of LiCl at all time points (Fig. 3I). We further evaluated the production of dsRNA in the SADS-CoV-infected cells, treated with or without LiCl, by confocal microscopy. Significant dsRNA was observed during SADS-CoV infection, but the amounts of dsRNA were markedly lower in the LiCl-treated cells compared to untreated cells (Fig. 3J). These results indicated that the GSK3 inhibitor results in a reduction in SADS-CoV RNA synthesis.

### GSK3 enhances NSP12 RdRp activity

**Fig 4:**
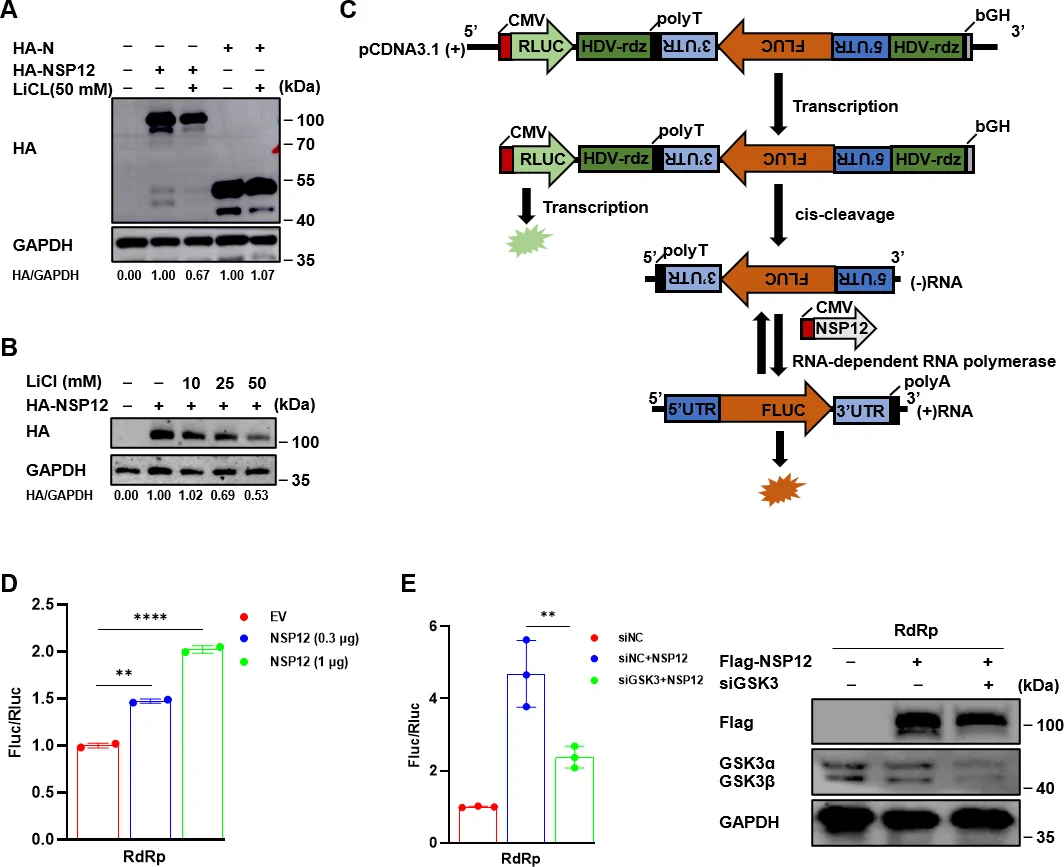
GSK3 enhances SADS-CoV RdRp-reporter activity. (**A**) LiCl disrupts the expression of NSP12 but not the N protein. The plasmids of NSP12 or N were transfected into Huh7 cells, respectively, for 24 h, followed by treatment with LiCl. At 24 hpt, the NSP12 and N protein levels were determined by western blotting. (**B**) The expression of the NSP12 protein treated with LiCl was reduced in a dose-dependent manner. (**C**) A schematic representation of a cell-based SADS-CoV RdRp activity assay is presented. (**D**) The SADS-CoV NSP12 protein is shown to activate RdRp activity. HEK293T cells were co-transfected with the pCDNA3.1-RdRp-reporter plasmid (1 µg) along with either an empty vector or pFlag-NSP12 at varying doses (0.3 or 1 µg) for 24 h. The firefly luciferase (Fluc) and Renilla luciferase (Rluc) activities were measured, and the ratio of Fluc to Rluc (Fluc/Rluc) was calculated to represent RdRp activity. (**E**) Knockdown of GSK3 significantly reduces SADS-CoV RdRp-reporter activity. HEK293T cells were transfected with either siNC or siGSK3 for 24 h, followed by transfection with NSP12 and the RdRp-reporter plasmids for an additional 24 h. RdRp activity was quantified as Fluc/Rluc. The expression levels of NSP12, GSK3α, and GSK3β were analyzed via western blotting using rabbit anti-Flag and mouse anti-GSK3 antibodies, respectively, with GAPDH serving as a loading control. Western blots were repeated at least three times, and one of them was selected as representative. Data are presented as mean ± SD, with statistical significance indicated as **P < 0.01 and ****P < 0.0001, determined by a two-tailed *t*-test.

Coronavirus RNA synthesis occurs within the replication-transcription complex (RTC), which comprises nonstructural proteins (NSPs) (45, 46). NSP12, which harbors the RNA-dependent RNA polymerase (RdRp) activity, is an essential component for coronavirus genome replication and RNA synthesis. As one of the highly abundant structural proteins in coronavirus, the N protein is also indispensable for coronavirus RNA synthesis (47). Previous studies reveal that the coronavirus N protein contains conserved consensus motifs that GSK3 phosphorylates, and inhibition of phosphorylated N protein disrupts viral template readthrough, resulting in a reduction of RNA synthesis (37–41). We subsequently detected the levels of SADS-CoV N and NSP12 proteins in the presence of a GSK3 inhibitor. No faster migrating bands or altered expression of the N protein were observed in the presence of LiCl. However, we observed that NSP12 protein levels were significantly decreased following LiCl treatment (Fig. 4A). Huh7 cells were transfected with pHA-NSP12 for 24 h, followed by treatment with LiCl at various concentrations for an additional 24 h. Western blotting results show that LiCl reduced NSP12 expression in a dose-dependent manner (Fig. 4B). As a critical component of RNA synthesis, the reduction of NSP12 levels may affect its RdRp activity. To verify our hypothesis, we constructed a cell-based SADS-CoV RdRp activity reporter system (SADS-CoV RdRp reporter) by modifying a similar previously established reporter system for HCV (43). In this reporter system, RdRp activity can be reflected by the ratio of *firefly* luciferase (Fluc) to *Renilla* luciferase (Rluc), with Rluc serving as the internal control (Fig. 4C). NSP12 binds to its cofactors NSP7 and NSP8 to form the RdRp complex, activating coronavirus RNA synthesis (48). However, NSP12 possesses intrinsic RdRp activity *in vitro* even in the absence of NSP7 and NSP8, and NSP12 alone is sufficient to activate the coronavirus RdRp reporter system (49–52). To determine whether SADS-CoV NSP12 drives Fluc expression, HEK293T cells were cotransfected with the RdRp reporter plasmid along with increasing doses of pFlag-NSP12. After 24 h, the activities of Fluc and Rluc were measured. As shown in Fig. 4D, a higher ratio of Fluc to Rluc (Fluc/Rluc) was observed in the NSP12-expressing cells in a dose-dependent manner, indicating that the SADS-CoV RdRp reporter was successfully constructed and was suitable for the following experiments. Using the established reporter system, we then determined the effect of GSK3 on SADS-CoV RdRp activity. HEK293T cells were transfected with siNC or siGSK3 for 24 h and subsequently cotransfected with NSP12 and the RdRp reporter plasmids for another 24 h. In the siNC-transfected cells, the Fluc/Rluc ratio was increased to 4.7-fold by NSP12 (Fig. 4E). However, knockdown of GSK3 significantly reduced this induction to only 2.4-fold (Fig. 4E). These data supported the role of GSK3 in enhancing SADS-CoV RdRp-regulated RNA synthesis.

### GSK3 interacts with NSP12

**Fig 5:**
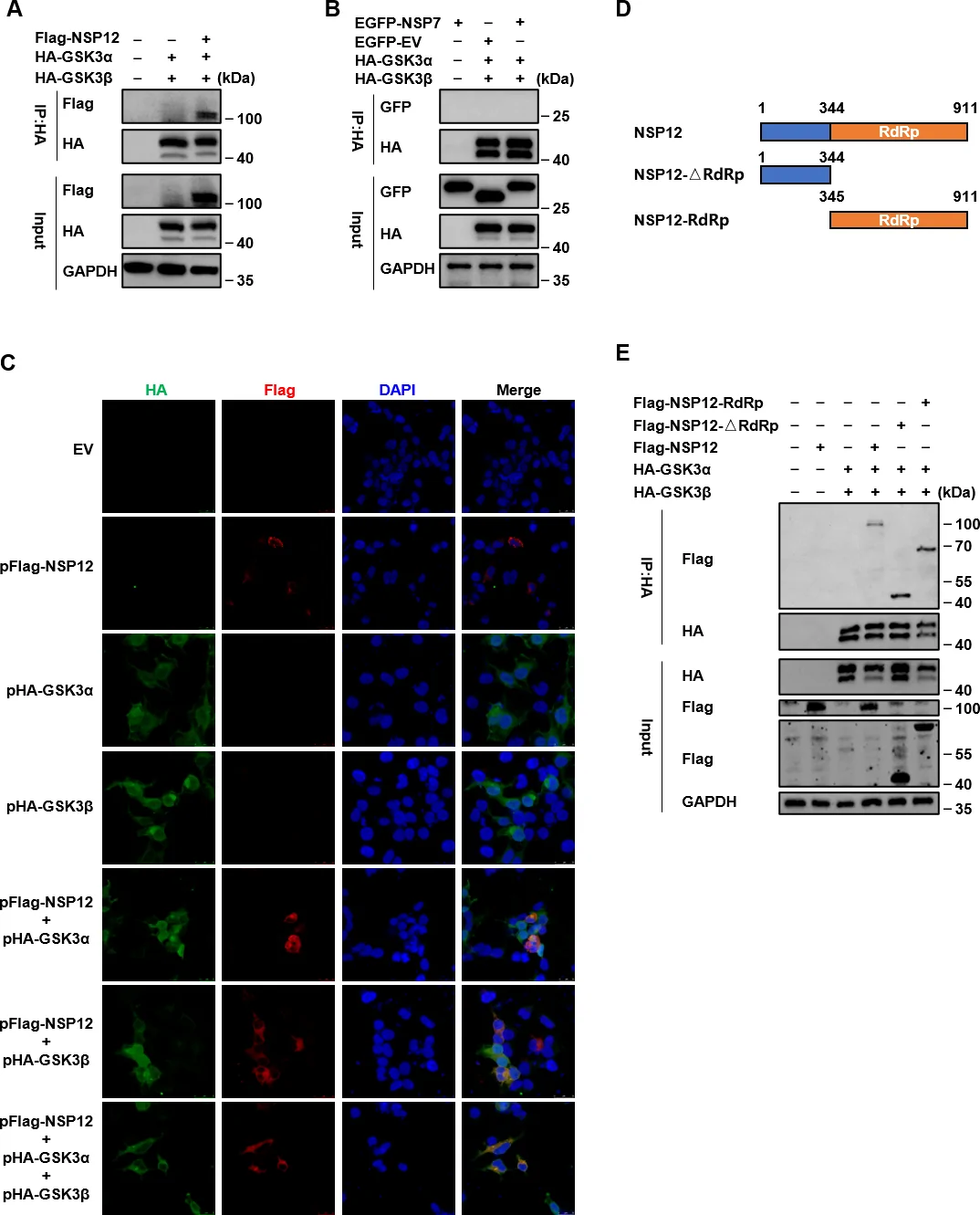
GSK3 interacts with the SADS-CoV NSP12. (**A and B**) HEK293T cells were cotransfected with pHA-GSK3 (GSK3α and GSK3β) and either an empty vector, pFlag-NSP12, or pEGFP-NSP7 for co-immunoprecipitation using HA-beads. (**C**) HEK293T cells were individually transfected with an empty vector, pFlag-NSP12, pHA-GSK3α, pHA-GSK3β, or cotransfected with pFlag-NSP12 alongside pHA-GSK3α and pHA-GSK3β for 36 h. The cells were then stained with mouse anti-Flag (red) and rabbit anti-HA (green) antibodies. Nuclei were stained with DAPI (blue), and their co-localization was detected using a confocal image. Scale bar, 25 µm. (**D**) A graphical representation of the NSP12 deletion construct is provided. (**E**) HEK293T cells co-expressing GSK3 and NSP12, NSP12-△RdRp, or NSP12-RdRp were immunoprecipitated with HA-beads. GAPDH as a loading control. Western blots were repeated at least three times, and one of them was selected as representative.

Overexpression of NSP12 alone induces SADS-CoV RdRp-reporter activity; however, the deficiency of GSK3 results in a reduction in reporter activity. We subsequently assessed the interaction between NSP12 and GSK3 using Co-immunoprecipitation (Co-IP). HEK293T cells were transfected with pHA-GSK3 (GSK3α and GSK3β) and either pFlag-NSP12 or pEGFP-NSP7 for 36 h. NSP12, but not NSP7, was detected in the GSK3-enriched samples, indicating that GSK3 interacted with NSP12 but not NSP7 (Fig. 5A and B). We subsequently analyzed the cellular distribution of NSP12 and GSK3 by expressing them either together or individually in the transfected cells. When expressed alone, both GSK3 and NSP12 localized to the cytoplasm. A significant colocalization of NSP12 and GSK3 was observed in the cytoplasm upon co-expression using confocal microscopy assay (Fig. 5C), a finding which further supported the Co-IP results. Given that the RdRp domain of NSP12 is catalytically central for the synthesis of viral RNA, we accordingly generated NSP12 truncated mutants either lacking the RdRp domain (NSP12-△RdRp) or only containing the core RdRp domain (NSP12-RdRp) (Fig. 5D). We transfected pFlag-NSP12, -NSP12-△RdRp, or -NSP12-RdRp with pHA-GSK3 into HEK293T cells, followed by Co-IP assay. Both mutants were detected in the HA-beads enriched materials (Fig. 5E). These results indicated that GSK3 specifically interacts with NSP12.

### GSK3 stabilizes NSP12 protein by inhibiting its proteasomal degradation

**Fig 6:**
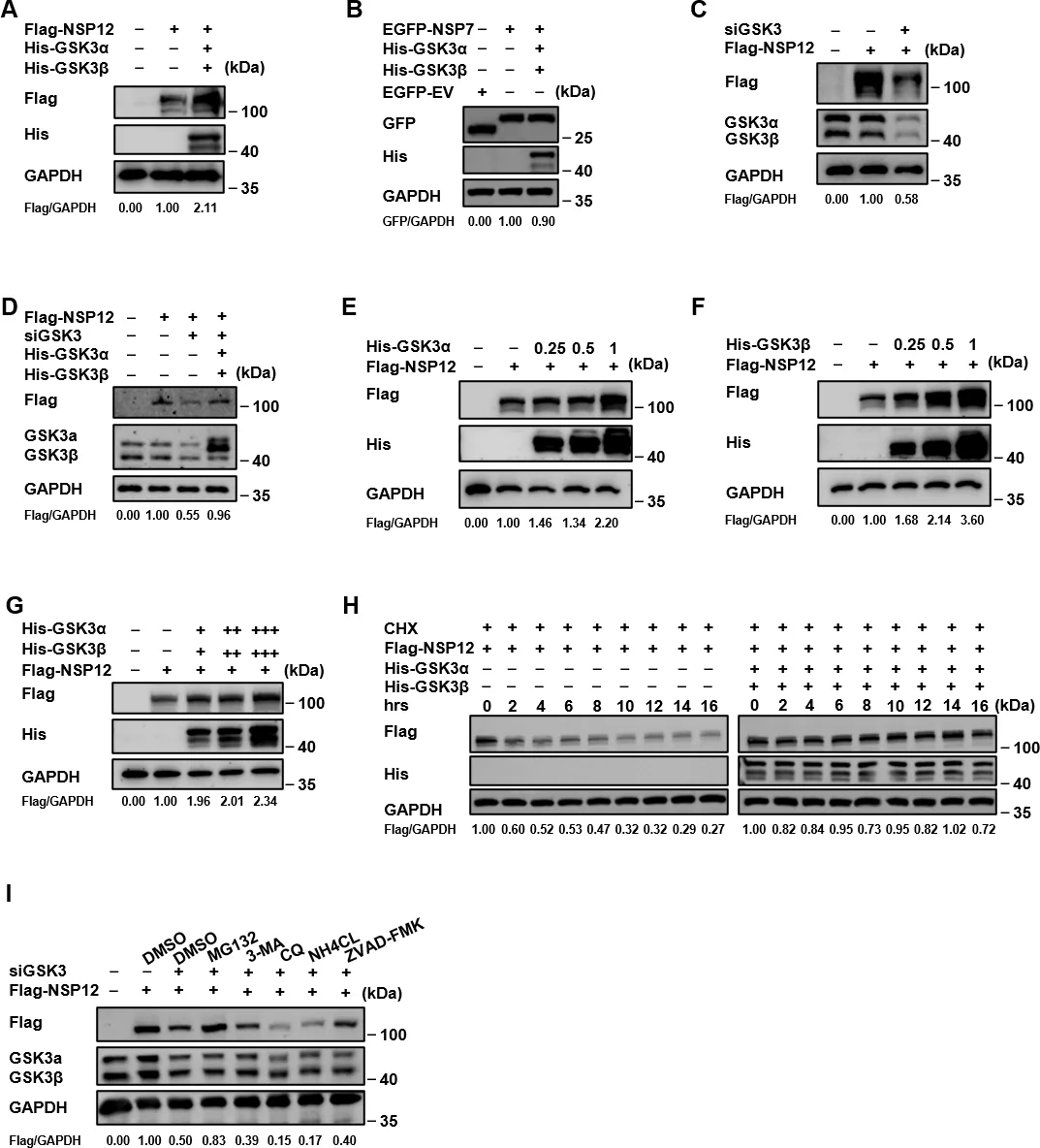
GSK3 stabilizes the NSP12 protein by blocking its proteasomal degradation. (**A and B**) GSK3 enhances the expression of the NSP12 protein but does not affect NSP7. (**C**) The NSP12 protein level in siGSK3 (siGSK3A and siGSK3B) transfected cells was determined at 24 h by western blotting using the indicated antibodies. (**D**) The re-expression of GSK3 in siGSK3-transfected cells restores the NSP12 protein level. (**E–G**) Two isoforms of GSK3 promote NSP12 protein expression in a dose-dependent manner. Cells were co-transfected with pFlag-NSP12 and pHis-GSK3α (**E**), pHis-GSK3β (**F**), or both isoforms (**G**) for 24 h, and the expression of pFlag-NSP12 and pHis-GSK3α/β was analyzed by western blotting. (**H**) An empty vector or pHis-GSK3 (GSK3α and GSK3β) and pFlag-NSP12 were cotransfected for 18 h, followed by treatment with cycloheximide (CHX) (200 µg/µL) for 0, 2, 4, 6, 8, 10, 12, 14, or 16 h. The NSP12 protein level was determined by western blotting. (**I**) pFlag-NSP12 was transfected into the GSK3 knockdown cells for 24 h and then treated for 12 h with DMSO, MG132 (20 µM), 3-MA (10 mM), CQ (50 nM), NH4Cl (25 mM), or ZVAD-FMK (10 µM), respectively. The expression levels of GSK3α/β and NSP12 were analyzed by western blotting using the indicated antibodies. GAPDH serves as a loading control. Western blots were repeated at least three times, and one of them was selected as representative.

Knockdown of GSK3 significantly attenuates SADS-CoV RdRp-reporter activity, accompanied by a reduction of NSP12 protein level, and GSK3 interacts with NSP12 (Fig. 4 and 5). We further evaluated the effect of GSK3 on the NSP12 protein level. Overexpression of GSK3 significantly increased NSP12 expression but not NSP7 (Fig. 6A and B). Conversely, GSK3 knockdown significantly reduced NSP12 expression, and this reduction was recovered by ectopic GSK3 expression (Fig. 6C and D). The two different GSK3 isoforms enhanced the expression of NSP12 in a dose-dependent manner (Fig. 6E through G). Subsequently, we cotransfected HEK293T cells with pFlag-NSP12 and pHis-GSK3 for 18 h, and then treated them with cycloheximide (CHX), which is a protein synthesis inhibitor. A significant reduction in NSP12 protein was observed at 2 hpt when NSP12 was expressed alone, whereas the expression level of the NSP12 protein was still stable at 16 hpt when both NSP12 and GSK3 were cotransfected (Fig. 6H). These results indicated that GSK3 maintained NSP12 stability. To understand the mechanism by which GSK3 stabilizes NSP12 expression, HEK293T cells were transfected with siNC or siGSK3 for 24 h and then transfected with pFlag-NSP12 for another 24 h. The cells were treated with the ubiquitin-proteasomal inhibitor MG132, the autophagy-lysosomal inhibitors 3-methyladenine (3-MA), chloroquine (CQ), or NH_4_CL, or the apoptosis inhibitor ZVAD-FMK for 12 h. Compared to siNC-transfected cells, the NSP12 protein level was sharply decreased in the siGSK3-transfected cells. However, the effect was effectively rescued by MG132 but not by autophagy-lysosomal inhibitors or an apoptosis inhibitor (Fig. 6I), indicating that GSK3 deficiency promoted the proteasomal degradation of the SADS-CoV NSP12 protein. Together, the results suggested that GSK3 increases NSP12 stability by inhibiting its proteasomal degradation.

### GSK3 promotes the deubiquitination activity of ubiquitin-specific peptidase 27X on the NSP12 protein

**Fig 7:**
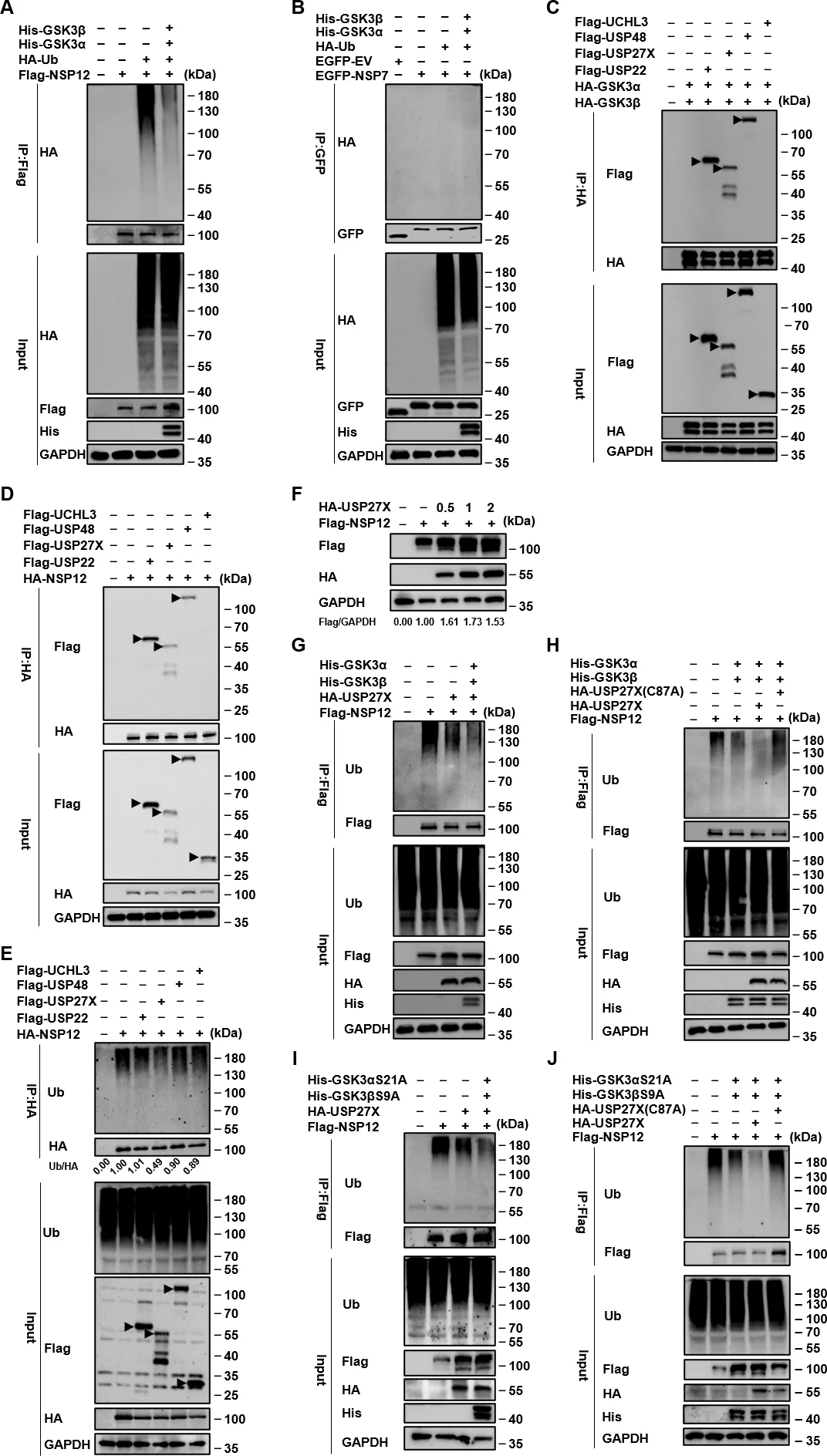
GSK3 enhances the deubiquitination activity of USP27X on the NSP12 protein. (**A**) GSK3 reduces the ubiquitination level of the NSP12 protein. HEK293T cells were co-transfected with pFlag-NSP12, pHA-Ubiquitin (Ub), and either pHis-GSK3 (GSK3α and GSK3β) or an empty vector for 24 h. Cell lysates were subjected to immunoprecipitation (IP) using Flag beads and analyzed by western blotting with the indicated antibodies. (**B**) The plasmids of pEGFP-NSP7, pHA-Ub, and pHis-GSK3, or an empty vector, were transfected into HEK293T cells. Cell lysates were immunoprecipitated using an anti-GFP antibody and protein A/G magnetic beads, and the ubiquitination level of NSP7 was determined by Western blotting. (**C**) GSK3 interacts with USP22, USP27X, and USP48. HEK293T cells were transfected with pHA-GSK3 (GSK3α and GSK3β) along with pFlag-USP22, pFlag-USP27X, pFlag-USP48, or pFlag-UCHL3. Cell lysates underwent IP with HA beads, followed by western blotting with antibodies against HA, Flag, and GAPDH. Black arrowheads indicate the bands corresponding to the target proteins. (**D**) NSP12 interacts with USP22, USP27X, and USP48. pHA-NSP12 was co-transfected with pFlag-USP22, pFlag-USP27X, pFlag-USP48, or pFlag-UCHL3 into HEK293T cells. After 24 h, cell lysates were immunoprecipitated using HA beads and analyzed by western blotting with antibodies against HA, Flag, or GAPDH. Black arrowheads indicate the bands corresponding to the target proteins. (**E**) USP27X deubiquitinates NSP12. pHA-NSP12 and pFlag-USP22, pFlag-USP27X, pFlag-USP48, or pFlag-UCHL3 were transfected into HEK293T cells for 24 h. The lysates were then subjected to IP with HA beads and analyzed by western blotting using antibodies against HA, Flag, Ub, or GAPDH. Black arrowheads indicate the bands corresponding to the target proteins. (**F**) USP27X enhances the protein level of NSP12. pFlag-NSP12 and pHA-USP27X were transfected into HEK293T cells at varying doses for 24 h. The NSP12 protein level was analyzed by western blotting. (**G**) GSK3 enhances the effect of USP27X on the deubiquitination of NSP12 protein. HEK293T cells were transfected with pFlag-NSP12, pHA-USP27X, and pHis-GSK3 (GSK3α and GSK3β). The cell lysates were subjected to immunoprecipitation with Flag beads, followed by analysis by western blotting with the indicated antibodies. (**H**) pFlag-NSP12, pHis-GSK3 (GSK3α and GSK3β), pHA-USP27X, or pHA-USP27X(C87A) were co-transfected into HEK293T cells, and the lysates were incubated with Flag beads. GAPDH serves as a loading control. (**I**) The constitutively active forms of GSK3α and GSK3β (GSK3αS21A and GSK3βS9A) plasmids, alongside pFlag-NSP12 and pHA-USP27X, were transfected into HEK293T cells, and the ubiquitination level of NSP12 was determined by western blotting. (**J**) pFlag-NSP12, pHis-GSK3αS21A and pHis-GSK3βS9A, pHA-USP27X, or pHA-USP27X(C87A) was co-transfected into HEK293T cells, and the lysates were incubated with Flag beads. The ubiquitinated NSP12 levels were determined by western blotting. GAPDH serves as a loading control. Western blots were repeated at least three times, and one of them was selected as representative.

Ubiquitination of substrates is an important posttranslational modification (PTM) involved in protein degradation. Cotransfection of NSP12 or NSP7 and GSK3 plasmids into HEK293T cells reduced the ubiquitination of NSP12, but not NSP7 (Fig. 7A and B). Although free monomeric HA-tagged ubiquitin has an expected molecular mass of approximately 10 kDa, the HA-reactive signals detected in the immunoblot primarily represent HA-ubiquitin covalently conjugated to cellular substrate proteins, rather than free HA-ubiquitin, confirming that the observed changes reflect ubiquitination of NSP12. However, the molecular mechanisms by which GSK3 regulates NSP12 ubiquitination remain unclear.

Protein ubiquitination is a dynamic process that is mainly regulated by E3 ubiquitin ligases or deubiquitinating enzymes (DUBs), which regulate protein degradation or stabilization, respectively. It has been reported that GSK3 interacts with and phosphorylates partial DUBs to enhance their deubiquitinating activity, or that GSK3-mediated phosphorylation of substrates enhances its interaction with DUBs, such as USP22, USP27X, USP48, or ubiquitin C-terminal hydrolase L3 (UCHL3), thereby facilitating further deubiquitination and stabilization of substrates (53–56). We found that USP22, USP27X, and USP48 interacted with GSK3 (Fig. 7C) or NSP12 (Fig. 7D), but only USP27X significantly reduced NSP12 ubiquitination, indicating that NSP12 is a substrate protein of USP27X (Fig. 7E). Overexpression of USP27X in HEK293T cells markedly increased NSP12 expression in a dose-dependent manner (Fig. 7F). USP27X contains a canonical GSK3 phosphorylated motif (S/TxxxS/T) and is phosphorylated by GSK3, which further enhances the deubiquitination activity of USP27X (54). We subsequently overexpressed GSK3α and GSK3β together with USP27X and NSP12 in HEK293T cells. Immunoblot analysis revealed that GSK3 further enhanced the USP27X-mediated deubiquitination of NSP12 (Fig. 7G). To assess the dependence of this effect on USP27X catalytic activity, we cotransfected GSK3 with either wild-type USP27X or its catalytically inactive USP27X mutant (USP27X-C87A). As shown in Fig. 7H, USP27X-C87A exhibited markedly diminished deubiquitinating activity and failed to reduce NSP12 ubiquitination. Phosphorylation of the N-terminal serine residues Ser21 of GSK3α and Ser9 of GSK3β represents the primary regulatory mechanism responsible for GSK3 inhibition. Mutations that block phosphorylation of these sites confer constitutive kinase activity (57, 58). Next, we overexpressed the constitutively active form of GSK3α (GSK3αS21A) and GSK3β (GSK3βS9A) together with USP27X and NSP12 in cells. Consistent with the results obtained with wild-type GSK3, GSK3αS21A and GSK3βS9A further reduced NSP12 ubiquitination in the presence of USP27X (Fig. 7I). However, constitutively active GSK3 mutants were not able to reduce the ubiquitination level of NSP12 when coexpressed with USP27X-C87A (Fig. 7J). Collectively, these findings demonstrate that GSK3 promotes NSP12 stabilization by potentiating the deubiquitinating activity of USP27X.

### GSK3 inhibitor protects neonatal mice against lethal SADS-CoV challenge

**Fig 8:**
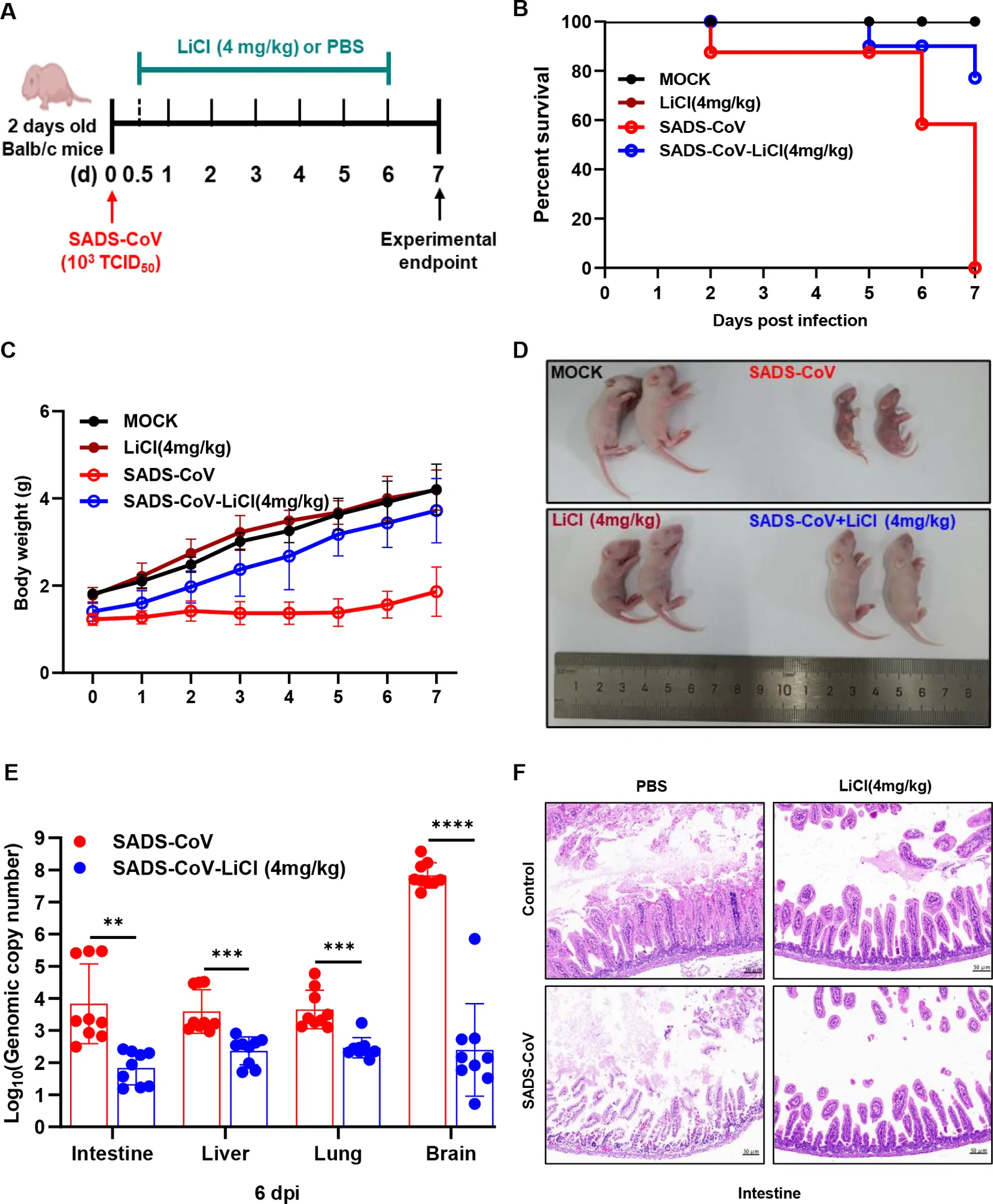
GSK3 antagonist protects neonatal mice against lethal SADS-CoV challenge. (**A**) A schematic representation of the neonatal mice experiment is presented. Two-day-old neonatal mice were divided into four groups. The mice were randomly assigned to the control group (*n* = 7), the LiCl-treatment group (4 mg/kg, *n* = 7), the SADS-CoV-infected group without LiCl treatment (*n* = 7), or the SADS-CoV-infected group treated with 4 mg/kg of LiCl (*n* = 8). The neonatal mice in the infected group were orally inoculated with 10^3^ TCID_50_ of the SADS-CoV GDS04 strain, while the other group received an equal volume of DMEM. After 12 h, the mice from the indicated groups were orally treated with 4 mg/kg of LiCl or an equal volume of PBS for 6 consecutive days. All mice were monitored for 7 days following the SADS-CoV challenge. (**B**) The survival rate of mice from each group was recorded until the end of the experiment. (**C**) Changes in body weight were monitored daily as described above. (**D**) The appearance of the body after infection with lethal SADS-CoV in neonatal mice is shown. (**E**) The viral load in the intestine, liver, lung, or brain from the SADS-CoV-infected group was determined by RT-qPCR at 6 days post-challenge. (**F**) Representative hematoxylin-eosin staining (HE) images of the intestine from each group are presented. Scale bar, 50 µm. Statistical significance is indicated as **P < 0.01, ***P < 0.001, and ****P < 0.0001, determined by a two-tailed *t*-test.

Ectopic GSK3 promoted SADS-CoV replication, while SADS-CoV replication was lower in GSK3α-KO or GSK3β-KO Huh7 cells than in the KO-CTRL cells. This indicated that drugs or inhibitors targeting GSK3 may have antiviral activity against SADS-CoV infection *in vivo*. Two-day-old neonatal mice were divided into four groups. Two groups were orally inoculated with 10^3^ TCID_50_ of SADS-CoV per mouse, and the other groups were orally administered an equal volume of DMEM as an uninfected group (Day 0). After 12 h post-challenge, the SADS-CoV-infected neonatal mice were orally treated with LiCl at 4 mg/kg or an equal volume of PBS for 6 consecutive days (Fig. 8A). We monitored the body weight change and survival rate of all neonatal mice daily for 7 days. As expected, all uninfected mice survived, and the neonatal mice challenged with SADS-CoV without LiCl treatment began to die at 2 dpi and all died within 7 days. However, the challenged neonatal mice treated with LiCl did not die until 5 dpi, and only two mice died by the end of the experiment, resulting in a survival rate of up to 75% (Fig. 8B). Notably, significantly greater weight loss was observed in untreated SADS-CoV-infected neonatal mice than in LiCl-treated or uninfected mice (Fig. 8C and D). Subsequently, we detected viral loads in the small intestine, liver, lung, and brain from SADS-CoV-challenged mice using RT-qPCR. The results showed a lower level of viral loads in the LiCl-treated neonatal mice than in the untreated neonatal mice (Fig. 8E). Histopathological analysis showed that the neonatal mice infected with SADS-CoV exhibited intestinal villous dissolution and reduced villus height. In contrast, the SADS-CoV-infected neonatal mice treated with LiCl showed restoration of intestinal villi structure and recovery of villus height (Fig. 8F). Together, these data suggested that the GSK3 inhibitor, LiCl, has potential as an antiviral agent for the management of SADS-CoV infection, particularly in the absence of an approved vaccine or antiviral drugs.

### GSK3 inhibitor suppresses PEDV, TGEV, and SADS-CoV infection

**Fig 9:**
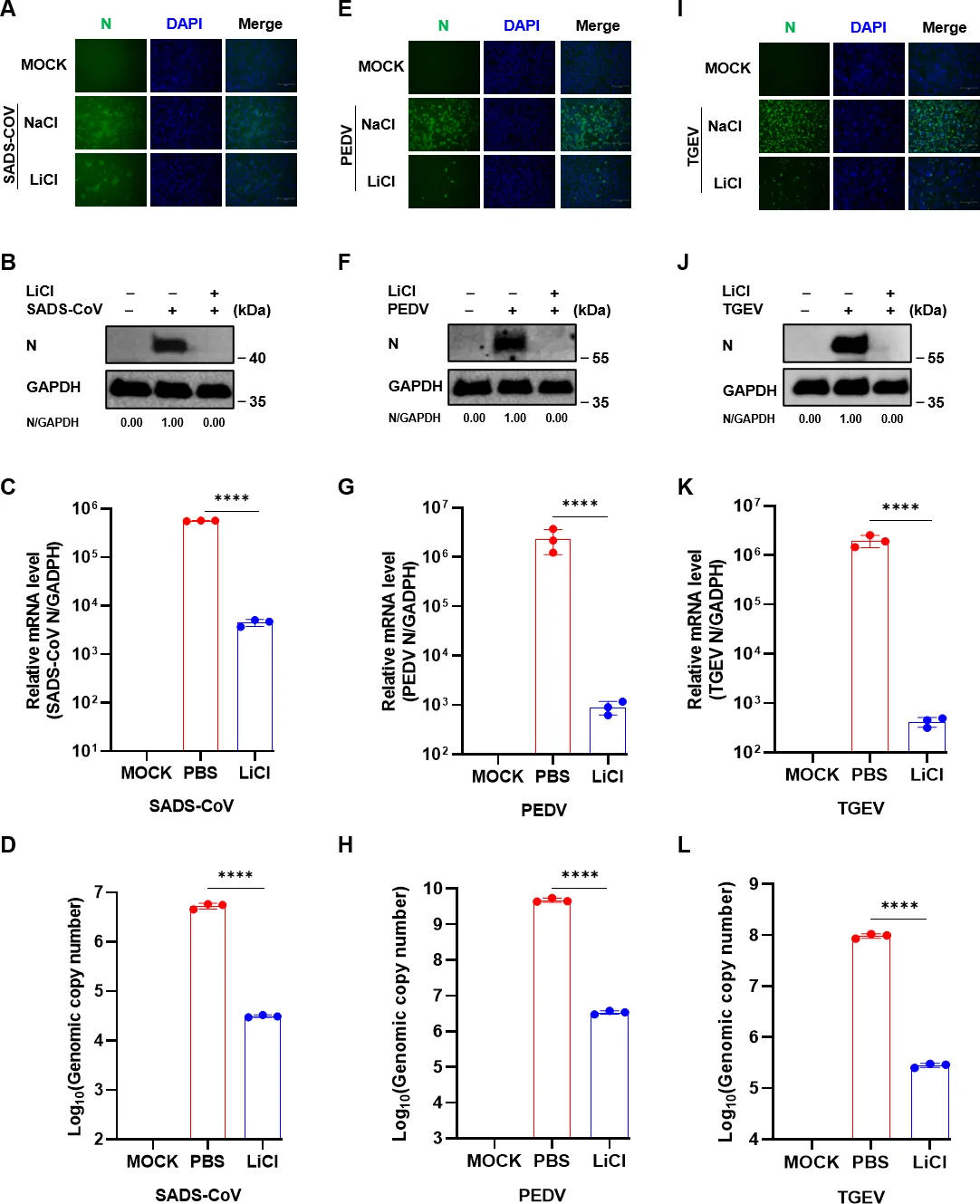
GSK3 inhibitor restricts swine enteric alphacoronaviruses infection. Huh7, Vero, or PK15 cells were infected with SADS-CoV, PEDV, or TGEV at a multiplicity of infection (MOI) of 1 for 2 h, followed by treatment with LiCl (25 mM). The replication of the virus was assessed using immunofluorescence assay (IFA) (**A, E, and I**) or western blotting (**B, F, and J**) with anti-N antibodies specific to SADS-CoV, PEDV, and TGEV, respectively. Scale bar, 300 µm. Western blots were repeated at least three times, and one of them was selected as representative. The transcription levels of the *N* gene (**C, G, and K**) and indicated viral genomic copy numbers (**D, H, and L**) were quantified using RT-qPCR. Data are presented as mean ± standard deviation (SD), with statistical significance indicated as ****, *P* < 0.0001, determined by a two-tailed *t*-test.

PEDV, TGEV, and SADS-CoV, which belong to the alphacoronavirus, are the main viral pathogens responsible for diarrheal disease in piglets. To explore the potential of GSK3 as a promising target for developing broad-spectrum antiviral drugs, we evaluated the infectivity of SADS-CoV, PEDV, or TGEV in Huh7, Vero, or PK15 cells treated with LiCl, respectively. Viral infectivity was determined by IFA, western blotting, and RT-qPCR, respectively. Huh7, PK-15, or Vero cells were infected with SADS-CoV, TGEV, or PEDV at an MOI of 1 for 2 h, followed by treatment with LiCl for 24 h. The viability of Vero and PK15 cells was not significantly affected by the presence of LiCl (Fig. S4A and B), but a significant reduction in N protein was observed in the SADS-CoV-infected cells (Fig. 9A and B), PEDV-infected cells (Fig. 9E and F), or TGEV-infected cells (Fig. 9I and J) in the presence of LiCl. Consistent with these findings, LiCl markedly inhibited viral replication as evidenced by significantly reduced viral genome copy numbers and decreased transcription levels of the respective *N* genes, as quantified by RT-qPCR (Fig. 9C, D, G, H, K, and L). Collectively, these results demonstrated that the GSK3 antagonist LiCl exhibits broad-spectrum antiviral activity against swine enteric alpha coronavirus infection (SADS-CoV, PEDV, and TGEV), further supporting the notion that GSK3 has great potential as a therapeutic target for the development of broad-spectrum anti-coronavirus agents.

## DISCUSSION

**Fig 10:**
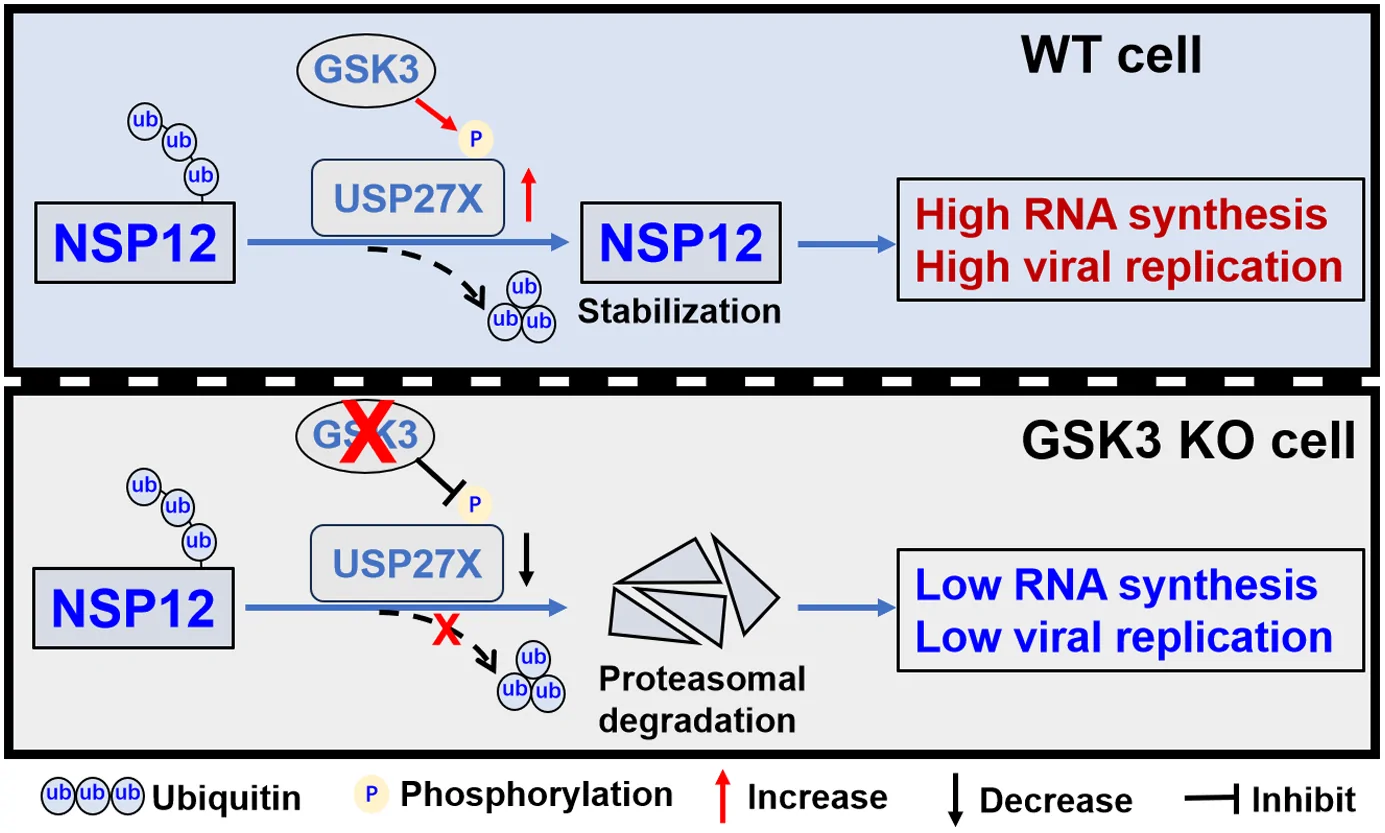
A model for GSK3 stabilizes the NSP12 protein by enhancing the deubiquitination activity of USP27X. NSP12, which harbors RNA-dependent RNA polymerase (RdRp), is a core catalytic component for RNA synthesis. In wild-type cells, GSK3 interacts with and enhances the deubiquitination activity of USP27X, thereby protecting the NSP12 protein from proteasomal degradation. This protection boosts viral RNA synthesis and replication. Conversely, GSK3 deficiency impairs the USP27X-mediated deubiquitination of the NSP12 protein, promoting its proteasomal degradation, which results in a reduction in viral RNA synthesis and replication.

Bat is an important reservoir host of viruses, some of which have spilled over to humans and resulted in fatal disease (59). The emergence of SADS-CoV indicates that the host range of bat-origin coronaviruses has expanded to pigs (15, 17, 19). Given the close contact between pigs and humans and the broad host cell tropism of SADS-CoV, its zoonotic transmission has attracted public attention. No licensed vaccines or drugs are available for SADS-CoV, and the knowledge of the host factors responsible for its infection remains poorly characterized. There is an urgent need to identify essential host dependency factors as potential targets for the development of broad-spectrum antiviral drugs, especially for emerging or re-emerging coronaviruses. Our study reveals that SADS-CoV exploits GSK3 to stabilize NSP12 by enhancing USP27X-mediated deubiquitination of NSP12, which protects NSP12 from proteasomal degradation and further facilitates viral RNA synthesis and replication (Fig. 10). Our findings uncover a novel mechanism underlying how GSK3 enhances SADS-CoV RNA synthesis by stabilizing the NSP12 protein and not only by phosphorylation of the coronavirus N protein as described previously (39).

GSK3, as a serine/threonine kinase, participates in the regulation of gene transcription, cellular metabolism, cytoskeleton reorganization, innate immune responses, and neurodegenerative diseases (34). Knockdown or knockout of GSK3 markedly inhibits SADS-CoV replication (Fig. 1; Fig. S1), but the stronger phenotype is observed in the knockdown condition than in the knockout condition. We speculate that this difference may reflect cellular adaptation or compensatory mechanisms in the knockout cells because knockout cells were selected over a longer period. By contrast, knockdown cells produce a more acute reduction of the target protein, which may reveal a stronger GSK3 defective effect before compensatory pathways emerge.

The deficiency of GSK3 or treatment with LiCl does not impair SADS-CoV attachment or internalization, but it significantly reduces viral dsRNA and suppresses the synthesis of both +vRNA and −vRNA (Fig. 2 and 3). Notably, GSK3 is recruited to and localized with dsRNA upon SADS-CoV infection (Fig. 2F). These results suggest that SADS-CoV may hijack GSK3 to facilitate its RNA synthesis. As shown in Fig. 2, dsRNA levels did not increase significantly in control cells, whereas the levels of positive- and negative-strand viral RNA continued to increase from 6 to 24 h. We speculate that because coronavirus dsRNA represents replication intermediates rather than total viral RNA, its signal can vary dynamically during infection. In addition, as viral replication progresses, dsRNA-containing replication intermediates may become sequestered within double-membrane vesicles, reducing their accessibility to antibody detection.

As a kinase, GSK3 phosphorylates the N protein of SARS-CoV or SARS-CoV-2 (37–41). The coronavirus N protein is a central structural protein essential for viral mRNA transcription and virion assembly (47). Phosphorylated N protein recruits RNA helicase DDX1 to facilitate viral discontinuous transcription and RNA synthesis. However, GSK3 inhibition disrupts N protein phosphorylation, resulting in the reduction of the longer subgenomic mRNA (sgmRNA) (39). Since both N protein phosphorylation and the RTC are critical for viral RNA synthesis, we assessed whether GSK3 modulates RTC function. Coronavirus transcription and replication are driven by NSP12, which acts as the core component of the RTCs. Although LiCl does not affect the migration or expression of the N protein, much lower NSP12 levels are detected (Fig. 4A and B). Moreover, knockdown of GSK3 severely impairs NSP12 protein expression, which is recovered upon ectopic GSK3 expression or treatment with a ubiquitin-proteasomal inhibitor (Fig. 6). An interaction between GSK3 and NSP12 was observed in our results (Fig. 5A and C), supporting the hypothesis that GSK3 regulates SADS-CoV replication by targeting NSP12. We tried to map the critical region of NSP12 that interacts with GSK3. We generated two NSP12 mutants (NSP12-RdRp and NSP12-△RdRp). However, the results showed that GSK3 interacted with both regions of NSP12 (Fig. 5E), suggesting that GSK3 may not bind NSP12 through a single linear motif but instead may interact with multiple regions of NSP12. One possible explanation is that NSP12 contains more than one GSK3-binding interface, with both the N-terminal region and the RdRp domain contributing to the stability of the GSK3-NSP12 complex. Such a multi-region interaction may help stabilize NSP12 and facilitate its functional association with GSK3. These results support the involvement of GSK3 in SADS-CoV RNA synthesis through maintaining NSP12 stability.

The reduction in NSP12 levels mediated by GSK3 deficiency is significantly recovered upon treatment with a ubiquitin-proteasomal inhibitor (Fig. 6I). The ubiquitination level of NSP12 is significantly decreased in GSK3-overexpressing cells (Fig. 7A). GSK3 is not a DUB, implying that GSK3 may cooperate with DUBs to facilitate NSP12 deubiquitination and thereby maintain its stabilization. It has been well documented that phosphorylation can regulate both the enzymatic activity and cellular localization of DUBs (60–62). Given that some DUBs are substrates of GSK3, GSK3 may influence their enzymatic activity or enhance the interaction between substrate and DUBs by regulating the phosphorylation of DUBs or substrate protein. A previous study indicated that GSK3 can enhance the ability of USP27X to remove the polyubiquitin chains, increasing the stability of the substrate protein (54). Our results also demonstrate that USP27X interacts with NSP12 and catalyzes the removal of polyubiquitin chains from NSP12 (Fig. 7D and E). Notably, co-expression of USP27X and GSK3 resulted in a significantly lower level of NSP12 ubiquitination compared to USP27X expression alone (Fig. 7G), indicating that GSK3 potentiates the deubiquitinating activity of USP27X toward NSP12.

The mutation, recombination, and evolution of coronaviruses frequently facilitate immune escape, thereby constraining the protective efficacy of currently licensed vaccines to provide protection, particularly against emerging or re-emerging coronaviruses. Therefore, the development of broad-spectrum antiviral drugs is particularly important. Both RdRp and M^pro^ are optimal antiviral drug targets, but drug resistance is a significant challenge for antiviral therapy. Remdesivir, an RdRp inhibitor, has a great antiviral effect on SARS-CoV, MERS-CoV, and SADS-CoV infection (63, 64). However, multiple reports demonstrate that SARS-CoV-2 has developed resistance to remdesivir (65) or an M^pro^ inhibitor (nirmatrelvir) (66). A targeted viral protein therapy may be difficult to achieve broad-spectrum activity and is prone to viral mutation escape and therapeutic resistance. Identifying critical host factors for virus replication may solve the problem and present a promising antiviral strategy. GSK3 enhances SADS-CoV NSP12 protein levels and further increases SADS-CoV replication. We also evaluate its potential as an antiviral drug target *in vitro* and *in vivo*. Two-day-old neonatal BALB/c mice are highly susceptible and lethal to SADS-CoV infection. However, this susceptibility shows a negative correlation with the mice’s age and is dependent on the route of viral inoculation (23, 24). We, therefore, evaluate the antiviral activity of a GSK3 inhibitor, LiCl, *in vivo* during SADS-CoV infection in 2-day-old BALB/C mice. While the LiCl dose of 4 mg/kg showed antiviral activity in the neonatal mouse model used in our study, it should not be directly extrapolated to adult mice. Previous studies in adult mice have generally employed LiCl doses exceeding 30 mg/kg (67–70), indicating that the 4 mg/kg dose used in our neonatal model is substantially lower than doses commonly evaluated in adult animals. Given the considerable physiological, metabolic, and immunological differences between neonatal and adult mice, the dose required to achieve biological effects may differ substantially between neonatal mice and adult mice. LiCl not only exhibits notable antiviral activity against SADS-CoV but also significantly inhibits PEDV and TGEV replication *in vitro* (Fig. 8 and 9), supporting that GSK3 is a possible target for the development of a broad-spectrum antiviral drug in the future. Both LiCl and CHIR-99021 inhibited SADS-CoV replication *in vitro*, and the present study focused on evaluating the antiviral activity of LiCl *in vivo*. Whether CHIR-99021 can similarly suppress SADS-CoV replication *in vivo* would be of interest and would provide additional insight into the role of GSK3 in SADS-CoV infection. Moreover, further studies are necessary to systematically evaluate the antiviral efficacy of the GSK3 inhibitor against prevalent strains and other viral-related diarrhea diseases in pigs.

Our study identifies GSK3 as an essential host factor for SADS-CoV replication and provides a novel mechanism by which GSK3 facilitates SADS-CoV RNA synthesis. GSK3 is recruited to viral dsRNA and stabilizes the NSP12 protein by enhancing the deubiquitinating activity of USP27X, which further increases SADS-CoV RNA synthesis. Notably, the GSK3 inhibitor exhibits a broad-spectrum antiviral effect against SADS-CoV, PEDV, and TGEV. Therefore, a comprehensive understanding of host factors involved in coronavirus RNA synthesis contributes to the development of antiviral therapeutics.

## Data Availability

All data supporting the conclusions of this study are available within the main text and supplemental material.

## SUPPLEMENTAL MATERIAL

Fig. S1 jvi.00683-26-s0001.tif Knockdown of GSK3 impairs SADS-CoV infection.

Fig. S2 jvi.00683-26-s0002.tif Knockout of GSK3ɑ or GSK3β does not influence cell viability.

Fig. S3 jvi.00683-26-s0003.tif Knockdown of GSK3 inhibits SADS-CoV double-stranded RNA (dsRNA) synthesis.

Fig. S4 jvi.00683-26-s0004.tif LiCl does not affect cell proliferation.

Supplemental legends jvi.00683-26-s0005.docx Descriptive legends for Fig. S1 to S4.

ASM does not own the copyrights to Supplemental Material that may be linked to, or accessed through, an article. The authors have granted ASM a non-exclusive, world-wide license to publish the Supplemental Material files. Please contact the corresponding author directly for reuse.
