# Supplementary material for: GSK3 maintains NSP12 stability to promote bat-origin swine acute diarrhea syndrome coronavirus infection
Source: J Virol. 2026 Sep 30;100(10):e00683-26. doi: 10.1128/jvi.00683-26 (PMC13712966; doi:10.1128/jvi.00683-26)
Supplement: Supplementary file 1 [file jvi.00683-26-s0005.docx]

**GSK3 maintains NSP12 stability to promote bat-origin swine acute diarrhea syndrome coronavirus infection**

Maowen Sun, Cong Yuan, Yueyue Duan, Lei Shi, Yu Zhang, Luyao Li, Wenquan Mao, Haixue Zheng*, Qi Wang*

State Key Laboratory for Animal Disease Control and Prevention, Lanzhou Veterinary Research Institute, Chinese Academy of Agricultural Sciences, Lanzhou, China.

* Corresponding author

E-mail: haixuezheng@163.com (HZ); qiwang@caas.cn (QW)

**Supplementary information**

**Figure S1.** **Knockdown of GSK3 impairs SADS-CoV infection.** (A and B) The knockdown efficacy of GSK3A and GSK3B was validated. Huh7 cells were transfected with 50 nM siRNA targeting GSK3A (siGSK3A-496, -635, or -1196) (A) or GSK3B (siGSK3B-1277, -1866, or -2073) (B) for 24 h. The mRNA levels of *GSK3A* and *GSK3B* were determined by reverse transcription-quantitative PCR (RT-qPCR). (C) The knockdown efficacy of siGSK3, a mixture of siGSK3A and siGSK3B, was validated. Huh7 cells were co-transfected with 30 nM siGSK3A-1198 and 50 nM siGSK3B-1277 (siGSK3). The mRNA levels of *GSK3A* and *GSK3B* were measured using RT-qPCR. (D and E) The knockdown of GSK3 suppresses SADS-CoV replication. Huh7 cells were transfected with siGSK3 for 24 h, followed by infection with SADS-CoV at a multiplicity of infection (MOI) of 1. The viral genomic copy number (D) and N protein level (E) were determined by RT-qPCR and western blotting at 24 hours post-infection (hpi), respectively. (F and G) The siGSK3A and siGSK3B have a great specificity. Huh7 cells were transfected with siGSK3A or si*GSK3B* for 24 h, and then the transcription level of *GSK3B* or *GSK3A* in the siGSK3A or siGSK3B-transfected Huh7 cells, respectively, was detected by RT-qPCR using the indicated primers. (H to J) The viral genomic copy numbers (H), titers (I), and N protein level (J), in siGSK3A, siGSK3B, or siGSK3 were determined by RT-qPCR, TCID50, and western blotting assays at 24 hpi, respectively. (K and L) Viral *N* transcription or N protein level in GSK3α- or GSK3β-expressed cell was determined by RT-qPCR (K) or western blotting (L) at 24 hpi, respectively. Western blots were repeated at least three times, and one of them was selected as representative. Data are presented as mean ± SD, with statistical significance indicated as ns, *P* > 0.05, ***P* < 0.01, ****P* < 0.001, and *****P* < 0.0001, determined by a two-tailed *t* test.

**Figure S2.** **Knockout of GSK3ɑ or GSK3β** **does not influence cell viability**. (A) KO-CTRL, GSK3ɑ-KO, or GSK3β-KO were cultured in 96-well plates for 24 h, and then CCK-8 solution was added to each well for another 4 h according to the manufacturer’s protocol. Measuring abundance at 450 nm. Data are shown as mean ± SD. ns, *P* > 0.05 by two-tailed *t* test.

**Figure S3.** **Knockdown of GSK3 inhibits SADS-CoV double-stranded RNA (dsRNA) synthesis.** (A and B) The knockdown of GSK3 (siGSK3) does not affect the attachment and internalization of SADS-CoV. Huh7 cells were pre-transfected with siGSK3 for 24 h, followed by incubation with SADS-CoV at an MOI of 1 for 1 h at 4°C (attachment assay) or subsequent incubation for an additional hour at 37°C (internalization assay). After three washes with PBS, total RNA was extracted to assess viral attachment (A) or internalization (B) using RT-qPCR. (C) siGSK3 disrupts the synthesis of viral dsRNA. Huh7 cells transfected with siGSK3 were infected with SADS-CoV (MOI = 1), and viral dsRNA (green) was stained using a mouse anti-dsRNA antibody at 8, 12, or 24 hpi. The quantification of viral dsRNA, performed using ImageJ software, is displayed in the bottom panel. Statistical significance is indicated as ns (*P* > 0.05) and ** (*P* < 0.01) based on a two-tailed *t* test.

**Figure S4.** **LiCl does not affect cell proliferation.** (A and B) Vero (A) or PK15 (B) cells were treated with different doses of LiCl at 0, 5, 10, or 25 mM. After 24 h, 10 µL of CCK-8 solution was added to 100 µL of cell medium for 4 h, followed by detection of absorbance at 450 nm according to the manufacturer’s protocol. Data are shown as mean ± SD. ns, *P* > 0.05 by two-tailed *t* test.
